# Interactions of the opioid and cannabinoid systems in reward: Insights from knockout studies

**DOI:** 10.3389/fphar.2015.00006

**Published:** 2015-02-05

**Authors:** Katia Befort

**Affiliations:** CNRS, Laboratoire de Neurosciences Cognitives et Adaptatives – UMR7364, Faculté de Psychologie, Neuropôle de Strasbourg – Université de Strasbourg, StrasbourgFrance

**Keywords:** opioid, cannabinoid, G protein-coupled receptors, reward, genetically modified mice

## Abstract

The opioid system consists of three receptors, mu, delta, and kappa, which are activated by endogenous opioid peptides (enkephalins, endorphins, and dynorphins). The endogenous cannabinoid system comprises lipid neuromodulators (endocannabinoids), enzymes for their synthesis and their degradation and two well-characterized receptors, cannabinoid receptors CB1 and CB2. These systems play a major role in the control of pain as well as in mood regulation, reward processing and the development of addiction. Both opioid and cannabinoid receptors are coupled to G proteins and are expressed throughout the brain reinforcement circuitry. Extending classical pharmacology, research using genetically modified mice has provided important progress in the identification of the specific contribution of each component of these endogenous systems *in vivo* on reward process. This review will summarize available genetic tools and our present knowledge on the consequences of gene knockout on reinforced behaviors in both systems, with a focus on their potential interactions. A better understanding of opioid–cannabinoid interactions may provide novel strategies for therapies in addicted individuals.

## INTRODUCTION

Drug abuse often leads to a complex pharmaco-dependent state which is defined by the term addiction. Addiction is considered as a neuropsychiatric disease. It develops from an initial recreational drug use, evolves toward compulsive drug-seeking behavior and excessive drug-intake with the appearance of negative emotional states such as anxiety or irritability when the drug is not accessible, and uncontrolled intake reaching a stage where the drug interferes with daily activities, despite the emergence of adverse consequences (Leshner, 1997; Everitt and Robbins, 2005; Robinson and Berridge, 2008; Koob, 2009). This pathological process develops in 15–30% of casual drug users and several factors may explain individual’s vulnerability to addiction, including genetic, psychological and environmental factors (Swendsen and Le Moal, 2011; Belin and Deroche-Gamonet, 2012; Pattij and De Vries, 2013; Saunders and Robinson, 2013). Addiction is a major threat to public health and represents a societal problem especially in developed countries and the economic cost it entails (investments in research, treatment and prevention) is considerable (Gustavsson et al., 2011).

Among illicit drugs, opiate and cannabinoid derivatives are highly abused in Europe. Morphine-like opiates are powerful analgesics and currently represent the major therapeutic remedies for the treatment of severe pain. They are also abused for their recreational euphoric effects. In Europe, 1.3 million people are addicted to heroin, the primary drug for which treatment requests are sought. Cannabis is the most worldwide consumed drug of abuse, with THC being the most abundant active constituent found in the various preparations of the drug. More than 73 million European citizens have used cannabis in the last year and it is estimated that about 7% of cannabis users has become dependent on this drug. There is also a high prevalence of users who seek treatment for dependence on it (http://www.emcdda.europa.eu/publications/edr/trends-developments/2014). Interestingly, new derivatives of these abused drugs are invading the market, notably through internet. Fentanyl derivatives as new opioid drugs and synthetic cannabimimetics, also known as “spices,” are becoming more and more popular (Fattore and Fratta, 2011). These abusive substances interact with two neuromodulator sytems, the opioid and the endocannabinoid systems.

### THE OPIOID SYSTEM

The opioid system consists of endogenous opioid peptides (enkephalins, endorphins, and dynorphins) from precursors (Penk, Pdyn, and Pomc) which activate three opioid receptors, namely mu, delta, and kappa (Kieffer, 1995). The three membrane receptors, cloned in the early nineties (Evans et al., 1992; Kieffer et al., 1992; Simonin et al., 1994, 1995; Mestek et al., 1995) are GPCR with coupling to Gi/Go proteins, of which the 3D structure was recently resolved (see Filizola and Devi, 2014). Opioid receptors and endogenous opioid peptides are largely expressed throughout the nervous system, noticeably within areas of the neurocircuitry of addiction associated with reward, motivation, or learning and stress (Mansour et al., 1995; Le Merrer et al., 2009; Koob and Volkow, 2010; Erbs et al., 2014). Besides its key role in many aspects of addition (Lutz and Kieffer, 2013a), the opioid system also plays a part in a diverse range of physiological functions including nociception, mood control, eating behavior, or cognitive processes (Contet et al., 2004; Pradhan et al., 2011; Stein, 2013; Bodnar, 2014; Nogueiras et al., 2014).

### THE ENDOCANNABINOID SYSTEM

**FIGURE 1:**
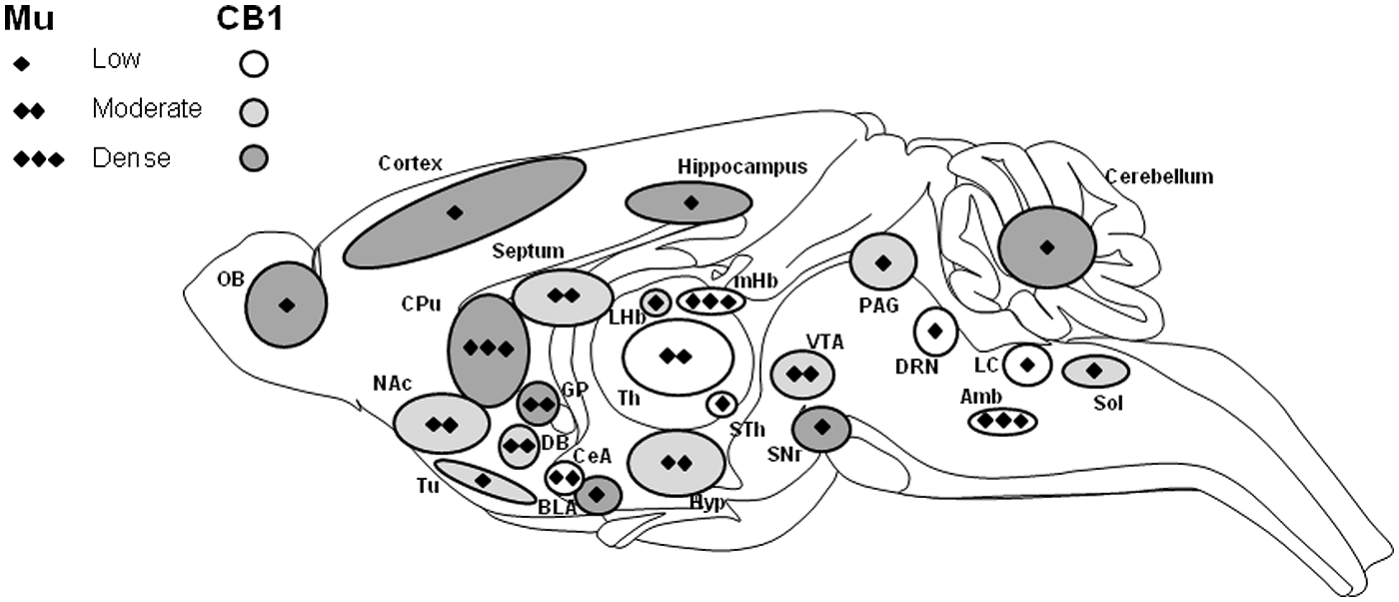
**Schematic representation of the distribution of mu opioid and CB1 cannabinoid receptors in the central nervous system.** CB1 receptor distribution over the whole central nervous system is indicated by circle shapes with low (white), moderate (gray) and high (dark gray) expression. Major localization of CB1 receptor (mRNA and protein) is in cortical areas, amygdala, striatum, and cerebellum. Moderate and low expression levels are observed in thalamic, hypothalamic, and brainstem regions. Interestingly, the mu opioid receptor is also expressed in these CB1 expressing brain areas but at various levels, indicated by diamonds for low (one), moderate (two), and high (three) expression levels (adapted from Mackie, 2005; Erbs et al., 2014 and references therein). Amb, ambiguous nucleus; BLA, basolateral amygdala; CeA, central amygdala; CPu, caudate putamen; DB, diagonal band; DRN, dorsal raphe nucleus; GP, globus pallidus; Hyp, hypothalamus; LC, locus coeruleus; LHb, lateral habenular nucleus; mHb, medial habenular nucleus; NAc, nucleus accumbens; OB, olfactory bulb; PAG, periaqueductal gray; SNr, substantia nigra pars reticulate; Sol, nucleus of the solitary tract; STh, subthalamic nucleus (ventral thalamus); Th, dorsal thalamus; Tu, olfactory tubercle; VTA, ventral tegmental area.

The endocannabinoid system is a neuromodulatory system consisting of two well characterized transmembrane receptors coupled to G protein (Gi/Go), CB1, and CB2 cloned in the 1990’s (Matsuda et al., 1990; Munro et al., 1993). The endogenous ligands are lipid neuromodulators, the main ones being AEA and 2-AG. Both are synthesized from phospholipid precursors and act locally as retrograde regulators of synaptic transmission throughout the central nervous system. These lipids are released by postsynaptic neurons and mainly activate presynaptic cannabinoid receptors to transiently or persistently suppress transmitter release from both excitatory and inhibitory synapses (recently reviewed in Ohno-Shosaku and Kano, 2014). Multiple pathways are involved in AEA biosynthesis with several still not fully characterized enzymes. AEA can be generated from the membrane phospholipid precursor N-arachidonoyl phosphatidylethalonamine (NAPE) through a two-step process with first a calcium-dependent transacylase followed by a phospholipase D (NAPE-PLD) hydrolysis (Liu et al., 2008). Phospholipase C (PLC) and DAGL are involved in 2-AG synthesis (Ahn et al., 2008). Their degradation is conducted by two specific enzymatic systems, the FAAH (Cravatt et al., 1996) and the MGL (Dinh et al., 2002), for AEA and 2-AG, respectively (Ahn et al., 2008). The endocannabinoid system plays a key role in energy balance, modulation of pain response, with processing of central and peripheral pain signals, learning and memory, reward and emotions. It has also been shown to be involved in neurogenesis and would play a neuroprotective role in some pathological conditions (for recent reviews see Gardner, 2005; Solinas et al., 2008; Maldonado et al., 2011; Zanettini et al., 2011; Panagis et al., 2014; Piomelli, 2014). Distribution of the two receptors in the central and peripheral system is rather different (Pertwee, 2010). Indeed, CB1 is highly abundant in the central nervous system in areas involved in reward, regulation of appetite and nociception (see **Figure 1**) while CB2 was initially described as a peripheral receptor (Maldonado et al., 2006, 2011; Mackie, 2008). Recent studies have proposed a low but significant expression of this receptor in several brain structures including striatum, hippocampus, and thalamus (Wotherspoon et al., 2005; Gong et al., 2006; Onaivi et al., 2006) and more recently into ventral tegmental area neurons (Zhang et al., 2014). Only few data are therefore available for the CB2 receptor in central function but growing evidence suggest a role in addictive processes, with an implication in cocaine, nicotine, or ethanol effects (Xi et al., 2011; Ignatowska-Jankowska et al., 2013; Navarrete et al., 2013; Ortega-Alvaro et al., 2013). To our knowledge, no data is available thus far concerning a potential role of CB2 in opioid mediated responses. Interestingly, other non-CB1 and non-CB2 receptors have been proposed to interact with endocannabinoids like the orphan GPCR GPR55 or a channel vanilloid TRPV1 recognizing capsaicin. These interactions could potentially explain some pharmacology of cannabis that cannot be accounted for by CB1 and CB2 activation, but further studies using KO approaches may help to provide a better understanding of this pharmacology (De Petrocellis and Di Marzo, 2010).

### CROSS TALK BETWEEN THESE NEUROTRANSMITTER SYSTEMS

Many neurotransmitter systems are involved when addiction develops, and both opioid and endocannabinoid systems are major players in addictive disorders. In addition to their specific ligands, both systems have also been implicated in the action mechanism of several other addictive drugs, like ethanol, nicotine, or psychostimulants. Although the endocannabinoid system has been known to interact with other systems like hypocretin, dopaminergic, and adenosinergic systems (Fernandez-Ruiz et al., 2010; Ferre et al., 2010; Tebano et al., 2012), its interaction with the opioid system is now well established (Fattore et al., 2005; Vigano et al., 2005; Robledo et al., 2008; Trigo et al., 2010). These two systems share neuroanatomical, neurochemical, and pharmacological, characteristics, this phenomenon is yet less well documented for the CB2 receptor. **Figure 1** illustrates brain structures expressing CB1 receptors and depicts expression level of mu opioid receptors in these areas. The existence of a specific opioid–cannabinoid interaction in the modulation of neurochemical effects as well as behavioral responses associated with reward and relapse have been demonstrated by pharmacological and genetic approaches but experimental results remain controversial (Manzanares et al., 1999; Fattore et al., 2005; Maldonado et al., 2011). Furthermore, molecular interactions between receptors have been shown with colocalization or heterodimerization data mainly for CB1 and delta or mu opioid receptors within spinal cord, striatum, or locus coeruleus. This phenomenon may also account for specific responses at the cellular level (Scavone et al., 2013; Massotte, 2014). However, the physiological effects of these molecular interactions have had yet to be revealed.

### AIM OF THE REVIEW

Pharmacological evidence for cross-talk with the synergetic effect of opioid and cannabinoid ligands in many functions related to addiction (mood, stress, learning process …) have been revealed and here we will review the implications of both systems regarding reward aspects. As several reviews have recently reported about these interactions (see above), we will focus our interest only on genetic studies using KO mice. We will first present the available genetic tools for both systems. We will then provide an update of results on reinforced behaviors to highlight insights into the particular role of the opioid system in responses to cannabinoids and the endocannabinoid system in responses to prototypical opiates like morphine. We will summarize the behavioral responses of KO mice to these drugs and propose a role for the potential interaction of these two endogenous systems in addictive processes.

## REWARD MEASURES IN MICE

Opioid and cannabinoid derivatives induce dependence. To study rewarding effects mediated by specific brain circuits in preclinical research, several behavioral models have been developed in rodents. The most reliable model to evaluate the reinforcing properties of a psychoactive compound in rodents is the self-administration (SA) paradigm which is based on a voluntary procedure to obtain the drug, coupled with the association of a signal (Panlilio and Goldberg, 2007). This operant system allows measuring both rewarding as well as motivational effects of an abused drug. Several aspect of addictive behaviors can be evaluated with this paradigm, with acquisition (fixed ratio) and motivation (progressive ratio) for the drug as well as extinction (response rate when drug-delivery has stopped) and reinstatement induced by cues, context or stress (relapse to drug-seeking) which will reflect aspects of excessive consumption (Sanchis-Segura and Spanagel, 2006). Intravenous SA has been extensively developed for opiates but more difficult to establish for cannabinoid compounds. Adaptations including drug priming, low doses, food restriction, animal restraint, or use of various cannabinoid agonists were often necessary (Maldonado, 2002; Panlilio et al., 2010; Panagis et al., 2014). Nevertheless, iv SA of both THC and the synthetic cannabinoid WIN55,212-2 have been successfully described both in rats and mice, and extended to the study of KO mice (Martellotta et al., 1998; Fattore et al., 2001; Mendizabal et al., 2006; Flores et al., 2014). A very recent study demonstrated for the first time that 2-AG is self-administered by rats and stimulates DA transmission (De Luca et al., 2014).

In addition, a well-accepted model to study the reinforcement properties of abused drugs is the CPP which is a non-operant paradigm. The reinforcing properties are associated with environmental stimuli (cues), such as the context in which the drug is administered. If the drug or a combination of drugs is aversive, animals avoid the drug-paired compartment (CPA) (Tzschentke, 2007). These paradigms have been widely used to study opiates or cannabinoids effects in mutant mice. However, data reporting reinforcing properties for THC and other cannabinoids are rather controversial with a critical concern about experimental conditions, with dose or injection schedule as major parameters to reveal either positive CPP or negative CPA properties of cannabinoids (Panagis et al., 2014).

On top of these two main paradigms (SA and CPP) other tasks have been developed like intracranial self-stimulation (ICSS) as a model to measure reward-facilitating effect of an abused substance although it is rather difficult to set up in mice and therefore little data is available (Panagis et al., 2014). Furthermore, withdrawal signs appear after cessation of chronic drug exposure, either spontaneously or precipitated by an antagonist treatment, and these signs can be scored for providing an index of dependence (Maldonado et al., 1996). In order to make a meaningful comparison in the evaluation of the specific involvement of components of opioid or cannabinoid systems in reward process, it is crucial to compare, when possible, the different mutant lines with their WT littermates in the exact same procedure to avoid bias from technical or experimental variations. Interestingly, such direct comparison has been recently performed for the four components of the opioid system (mu, delta, Penk, and Pdyn) to demonstrate differential behavior in the acquisition and relapse of cocaine SA in the four mutant mice (Gutierrez-Cuesta et al., 2014).

## GENERATION OF DEFICIENT MICE IN REGARDS TO COMPONENTS OF THE OPIOID OR CANNABINOID SYSTEMS

For each component of the opioid and the cannabinoid systems, various lines of genetically modified mice have been generated. **Table 1** presents a list for conventional KO mouse lines that have been described so far. The original papers describing the development of the constitutive deletion are presented with the targeted area of the suppressed gene.

**Table 1:** Knockout mouse lines for the opioid and the cannabinoid systems.

*Gene knockout*	Targeted exon	Reference
***Opioid system***
*Oprm*	Exon 2	Matthes et al. (1996)
	Exon 1	Sora et al. (2001)
	Exon 1	Tian et al. (1997)
	Exons 2 and 3	Loh et al. (1998)
	Exon 1	Schuller et al. (1999)
	Exons 2 and 3	van Rijn and Whistler (2009)
	Exon 11 (splice variant)	Pan et al. (2009)
*Oprd*	Exon 2	Zhu et al. (1999)
	Exon 1	Filliol et al. (2000)
	Exon 2	van Rijn and Whistler (2009)
*Oprk*	Exon 1	Simonin et al. (1998)
	Exon 3	Hough et al. (2000)
	Exon 3	Ansonoff et al. (2006)
	Exon 2	van Rijn and Whistler (2009)
	Exon 3	Van’t Veer et al. (2013)
*Oprm/oprd*		Simonin et al. (2001)
*Oprm/oprd/oprk*		Simonin et al. (2001)
		Clarke et al. (2002)
*Penk*	Exon 3	Konig et al. (1996)
	Exon 3	Ragnauth et al. (2001)
*Pdyn*	Exon 3	Sharifi et al. (2001)
	Exon 3	Zimmer et al. (2001)
	Exon 3	Loacker et al. (2007)
*Pomc*	Exon 3	Rubinstein et al. (1996)
	Exon 3	Yaswen et al. (1999)
*Penk/Pdyn*		Clarke et al. (2003)
***Cannabinoid system***
*Cnr1*	Exon 2	Zimmer et al. (1999)
	Exon 2	Ledent et al. (1999)
	Exon 2	Marsicano et al. (2002)
	Exon 2	Robbe et al. (2002)
*Cnr2*	Exon 2	Jarai et al. (1999), Buckley et al. (2000)
	Exon 2	Wotherspoon et al. (2005)
*FAAH*	Exon 1	Cravatt et al. (2001)
*MGL*	Exon 3	Uchigashima et al. (2011)
	Exons 3 and 4	Taschler et al. (2011)
	Intron 3–exon 4 (gene trapping)	Schlosburg et al. (2010)
	Exons 1 and 2	Chanda et al. (2010)
NAPE-PLD	Exon 4	Leung et al. (2006)
	Exon 3	Tsuboi et al. (2011)
*DAGLalpha*	Exon 1	Gao et al. (2010)
	Exons 3 and 4	Tanimura et al. (2010)
	Intron 4-Exon 1(gene trapping)	Yoshino et al. (2011)
*DAGLbeta*	Exon 1	Gao et al. (2010)
	Exons 10 and 11	Tanimura et al. (2010)
	Exon 1 (gene trapping)	Yoshino et al. (2011)
*cnr1/cnr2*		Jarai et al. (1999)
*FAAH/cnr1*		Sun et al. (2009)
*FAAH/cnr2*		Sun et al. (2009)
*FAAH/cnr1*		Wise et al. (2007)

This table summarizes the published report of KO mouse lines for the different partners of these two systems and combinatorial lines, with the original papers as reference. The area of the gene that has been targeted is indicated.

### THE OPIOID SYSTEM

For components of the opioid system, the mu receptor drew the most attention with the description of six distinct genetically modified lines targeting the coding regions of the *oprm* gene, with either exon 1, exon 2 or both exons 2 and 3 targeted for the deletion (Matthes et al., 1996; Tian et al., 1997; Loh et al., 1998; Schuller et al., 1999; Sora et al., 2001; Pan et al., 2009; van Rijn and Whistler, 2009). Interestingly, the mu opioid receptor KO mice allowed to unambiguously demonstrate that the mu receptor was the molecular target for morphine, the prototype of opiate ligand widely used in clinics for its therapeutic effect in pain relief. Morphine had neither analgesic effects nor rewarding properties in these mutant mice (for reviews, see Contet et al., 2004; Gaveriaux-Ruff, 2013). An additional mutant line was constructed which targeted exon 11, a splice variant for the mu receptor, located upstream of exon 1. In these deficient mice, a 25% decrease of receptor expression was observed (Pan et al., 2009), leading to difficult interpretation of the KO effect on opiate pharmacology (Gaveriaux-Ruff, 2013). For deletion of the delta receptor, either exon 1 or 2 were targeted in the *oprd* gene (Zhu et al., 1999; Filliol et al., 2000; van Rijn and Whistler, 2009). These mice were characterized for behavioral responses related to mood and analgesia, but the contribution of delta receptor in reward processes was less clear (Pradhan et al., 2011; Charbogne et al., 2014). Five distinct constructions have been reported targeting either exon 1, 2, or 3 of the *oprk* gene to obtain KO mice for the kappa opioid receptor (Simonin et al., 1998; Hough et al., 2000; Ansonoff et al., 2006; van Rijn and Whistler, 2009; Van’t Veer et al., 2013). The two most recent mutants were strategically obtained in order to generate a parallel conditional KO mice (see below) using a Cre-lox approach, with targeted exons floxed with loxP sites. The mutation impaired pharmacological actions of the selective kappa-agonist U-50,488H, and revealed a tonic implication of kappa receptors in the perception of visceral pain. Morphine-CPP was unchanged, but both morphine withdrawal signs as well as emotional responses during opiate abstinence were reduced (Simonin et al., 1998; Lutz et al., 2014), suggesting an anti-reward role for kappa receptors.

Mice with deleted opioid peptide precursors were also generated. For proopiomelanocortin (*Pomc*), two lines were produced, one specifically deleting βendorphin (Rubinstein et al., 1996) while the second was targeting the whole coding region, deleting both opioid and non-opioid active petides (Yaswen et al., 1999). KO mice for *Penk* gene were generated by two distinct laboratories, both leading to deletion of the 5^′^ part of exon 3 (Konig et al., 1996; Ragnauth et al., 2001). For deleting dynorphin in mutant animals, exons 3 and 4 (Sharifi et al., 2001) or exon 3 with a part of exon 4 (Zimmer et al., 2001) of the *Pdyn* gene were targeted. Data from peptide KO mice in regards to opiate rewarding effect were more complex. The βendorphin KO mice showed increased (Skoubis et al., 2005) or unchanged (Niikura et al., 2008) morphine-CPP depending on the dose and paradigm used and it was invariable both in mice lacking Penk (Skoubis et al., 2005) or Pdyn (Zimmer et al., 2001; Mizoguchi et al., 2010).

### THE CANNABINOID SYSTEM

Four independent KO lines have been generated for the CB1 receptor, encoded by a single large coding exon in the *cnr1* gene (exon 2). The first three lines were generated with the introduction of a PGK or neomycine resistance cassette in the coding region (Ledent et al., 1999; Zimmer et al., 1999; Robbe et al., 2002). For the fourth line, loxP sites were introduced flanking exon 2 and this floxed line was further crossed with a line constitutively expressing the Cre recombinase enzyme, therefore generating a full CB1 KO by deletion of the sequence between the two lox P sites (Marsicano et al., 2002). These mice were mostly unresponsive to cannabinoid ligands in mediating analgesia, reinforcement, hypothermia, hypolocomotion, and hypotension (Valverde and Torrens, 2012; Nadal et al., 2013). Two mouse lines were described for the deletion of the *cnr2* gene coding for CB2 receptor, one by Zimmer’s team (Jarai et al., 1999; Buckley et al., 2000) and the other one by the company Deltagen (Wotherspoon et al., 2005). Both were developed by deleting part of the coding region (in exon 2), leaving the start codon with a portion of the amino terminus sequence and aminoacids coding for some transmembrane domains of the receptor. In these constructions expression of the amino-terminal part of the CB2 receptor could potentially occur, but in both cases, it was shown that the receptor was non-functional in the mutant mice (Dhopeshwarkar and Mackie, 2014). Two mutant lines have been described for the NAPE-PLD enzyme involved in AEA synthesis, targeting exon 3 (Tsuboi et al., 2011) or exon 4 (Leung et al., 2006). These KO mice have highlighted the complexity of AEA synthesis with both calcium-dependent and -independent mechanisms. Two isoforms of DAGLα and DAGLβ responsible for the synthesis of 2-AG have been described and KO lines have been generated for each of them with both homologous recombination and gene trapping approaches (Gao et al., 2010; Tanimura et al., 2010; Yoshino et al., 2011). The DAGLα KO animals showed a markedly reduced 2-AG brain content whereas levels were normal in brain regions of KO for the β isoform indicating a much greater contribution of DAGLα to 2-AG biosynthesis in the central nervous system. These mutant mice were particularly useful in the characterization of DAGL involvement in retrograde endocannabinoid signaling (Frazier, 2011). The endocannabinoid system is characterized by a rapid catabolism of the endogenous ligands. Among the degrading enzymes of endocannabinoids, FAAH is the major enzyme responsible for the degradation of AEA and one KO line was generated targeting exon 1 of the *Faah* gene (Cravatt et al., 2001). These mutant mice exhibited more than 15-fold higher brain levels of AEA than WT animals and displayed reduced pain sensitivity. The major degrading enzyme of the endocannabinoid 2-AG is MGL and four KO lines were generated. Three KO lines targeting *mgll* gene exons 1 and 2 (Chanda et al., 2010), exon 3, or exons 3 and 4 were recently generated with a Cre/lox approach (Taschler et al., 2011; Uchigashima et al., 2011). Another line was obtained by gene trapping technology (Texas Institute of Genomic Medicine) with a gene trap cassette inserted into the *mgll* intron 3, upstream of the catalytic exon 4 (Schlosburg et al., 2010). Genetic deletion of MGL leads to alteration in endocannabinoid signaling with increased brain 2-AG levels by ∼10-fold. These animals were mainly characterized by behavioral consequences of the gene deletion for pain perception (Schlosburg et al., 2010; Uchigashima et al., 2011; Petrenko et al., 2014).

### COMBINATORIAL MOUSE LINES

Interbreeding of mutant mouse lines allowed generating combinatorial mutant mice both within the opioid and the cannabinoid systems (see references in **Table 1**). These combinatorial lines constituted useful tools to clarify the specific role of particular components of both systems in reward and analgesia, as well as to evaluate *in vivo* selectivity for specific ligands and receptor subtype identification (Kieffer and Gaveriaux-Ruff, 2002; Nadal et al., 2013). Data for reward responses obtained using multiple mutants for cannabinoid or opioid components are detailed below.

### COMPENSATORY EFFECTS OF THE NULL MUTATION

Globally, a normal development was described for the various mutant lines, with KO mice being fertile, caring for their offspring, and not showing any major behavioral abnormalities. A higher mortality rate was described for one of the CB1 KO line (Zimmer et al., 1999) but not reported for the two others. Interestingly, among the combinatorial mice, the triple mutant of the opioid receptors present a striking increase in body weight and size, but this obese-like phenotype needs further characterization (Befort and Gaveriaux-Ruff, personal communication). Compensatory mechanisms may have developed in some KO animals, but no systematic studies are available. Deletion of opioid receptors did not markedly modify the expression or activity of the other opioid receptors or the expression of opioid peptides as described by the initial characterizations of the distinct mutants lines (see references in **Table 1** and Kitchen et al., 1997; Slowe et al., 1999; Oakley et al., 2003). A complete autoradiographic mapping of the delta KO mice indicated decreased binding levels of mu and kappa ligands in specific brain areas (Goody et al., 2002). Deletion of opioid peptides modified other partners of the opioid system, with a region-dependent increased of both mu and delta receptor expression levels observed in the Penk KO line (Brady et al., 1999; Clarke et al., 2003) and for the three opioid receptors in the Pdyn and the double Pdyn/Penk mutant line with no additive effects (Clarke et al., 2003). Interestingly, specific changes of CB1 receptor expression or activity were reported in mu and delta opioid receptor mutant lines (Berrendero et al., 2003). In the mu KO brain, there was no difference in CB1 expression but a decreased efficacy of the classical cannabinoid agonist WIN 55,212-2 was observed specifically in the CPu while both density of CB1 receptor and activation by WIN 55,212-2 increased in substantia nigra of delta KO animals.

Compensatory effects in KO animals concerning the cannabinoid system have been described both for receptor or catabolic enzyme KO mice. The invalidation of the CB1 receptor gene was associated with age-dependent adaptive changes of endocannabinoid metabolism, with increased FAAH and AEA membrane transporter activities in KO hippocampus and cortex, decreased AEA content in hippocampus but no change in 2-AG levels (Di Marzo et al., 2000; Maccarrone et al., 2001, 2002). In the FAAH KO mice, CB1 receptor mRNA decreased in CPu, nucleus accumbens (core), hippocampus (CA1), hypothalamic nucleus (VMN), and amygdala. Its functional activity was also markedly reduced in CPu, the core of nucleus accumbens, and CA3 region of the hippocampus (Vinod et al., 2008). Interestingly, reduction of CB1 receptor density and activity were also observed in MGL KO mouse brain, which may prevent the manifestation of the dramatically enhanced 2-AG behavioral effects in these mice (Chanda et al., 2010; Schlosburg et al., 2010). In DAGLα- and DAGLβ-KO, no difference was reported for CB1 mRNA (Gao et al., 2010) or protein (Tanimura et al., 2010) levels in comparison to WT mice. In these KO mice, CB1 brain functional signaling was unaltered (Aaltonen et al., 2014). To our knowledge, no data is available for any compensatory effect on CB2 expression or activity in the distinct cannabinoid KOs. However, some reports indicate modifications of the opioid system in CB1 KO animals. An increase of both enkephalin and dynorphin mRNA expression was observed in the striatum (Steiner et al., 1999; Gerald et al., 2006, 2008) as well as an increase in kappa and delta opioid receptor activities without changes in their binding (Uriguen et al., 2005). No compensatory changes of mRNA levels for the three opioid receptors were reported in dorsal root ganglia or spinal cord of the CB1 KO animals (Pol et al., 2006). In FAAH KO mice, Penk mRNA expression was decreased in both CPu and nucleus accumbens which paralleled a reduced mu opioid receptor functional activity (Vinod et al., 2008). Noteworthy, these compensatory alterations of opioid or cannabinoid components in specific regions of the mutant lines could account for interactions of the two systems which may be relevant for neuroadaptative processes involved in drug dependence.

### CONDITIONAL APPROACHES

Knockout mice are very useful tools for understanding the contribution of each component of these systems in various conditions including pain, mood disorders or addiction (Valverde and Torrens, 2012; Gaveriaux-Ruff, 2013; Lutz and Kieffer, 2013b; Nadal et al., 2013; Charbogne et al., 2014). Recent approaches using gene manipulation in mice have been developed with the widely used Cre-loxP recombinase system to generate cKO (Fowler and Kenny, 2012; **Table 2**). It consists of crossing mice whose target genes are floxed (flanked with two loxP sites) together with mice expressing the Cre-recombinase under a specific promoter. This allows a time-, organ- or site-specific deletion of a target gene. This strategy allowed uncoupling the central and peripheral functions of CB1 receptors (Agarwal et al., 2007) and more recently of mu or delta opioid receptors (Gaveriaux-Ruff et al., 2011; Weibel et al., 2013) using the promoter of the channel Nav1.8 only expressed in DRGs, revealing a key role for these receptors expressed in primary nociceptive neurons in inflammatory pain. To investigate molecular mechanisms at the level of neuronal circuitry, selective deletion of a particular gene can also be achieved in specific neuronal types. For example, deletion of the delta opioid receptors specifically in forebrain GABAergic neurons was obtained by crossing a delta opioid floxed mouse line (Gaveriaux-Ruff et al., 2011) together with a dlx5-6-Cre mouse line, specifically expressing the Cre-recombinase in GABAergic forebrain neurons in order to investigate the role of these specific delta receptors in anxiety (Chu Sin Chung et al., 2014). This latter mouse line was previously crossed with the CB1 floxed mice to successfully obtain a GABA-CB1 conditional mutant (Monory et al., 2006). These mutants were also compared with several other cKO bearing a deletion of CB1 receptor in differing specific neuronal populations: forebrain glutamatergic neurons (CB1CamKIIa-Cre mice or CaMK-CB1KO), cortical glutamatergic neurons (CB1NEX-Cre mice or Glu-CB1KO), both glutamatergic and GABAergic neurons (Glu/GABA-CB1KO) or D1-dopaminergic neurons (CB1Drd1a-Cre mice) (Marsicano et al., 2003; Monory et al., 2006, 2007; Bellocchio et al., 2010) for studying the role of CB1 receptors as well as behavioral and autonomic effects of the agonist THC. For the opioid system, a recent study reported the generation of a conditional mutant for the kappa opioid receptor, selectively deleted in DA-expressing neurons. These kappa cKO mice showed reduced anxiety-like behavior as well as increased sensitivity to cocaine, consistent with a role for kappa receptors in negative regulation of DA function (Van’t Veer et al., 2013). For the cannabinoid system, cKO lines were also generated for the CB1 receptor to study its specific implication in neurons (Maresz et al., 2007) or peripheral nerves (Pryce et al., 2014), in serotoninergic (Dubreucq et al., 2012b) or paraventricular (Dubreucq et al., 2012a) and ventromedial (Bellocchio et al., 2013) hypothalamic neurons. CB1 was also specifically deleted in astroglial cells to investigate its role in working memory and long-term hippocampal depression (Han et al., 2012). CB1 was deleted in specific cell types like hepatocytes to study its role in ethanol-induced fatty liver (Jeong et al., 2008), lymphocytes (Maresz et al., 2007) or epidermal keratinocytes (Gaffal et al., 2013) to investigate its potential role in regulation of inflammatory responses. Another strategy to generate a cKO mouse is by using viral mediated construct carrying the Cre-recombinase injected directly in the structure of interest of a target gene-floxed mouse. For example, the mu opioid receptor was selectively deleted in the dorsal raphe, the main serotoninergic brain area, and this deletion abolished the development of social withdrawal in a model of heroin abstinence (Lutz et al., 2014).

**Table 2:** Conditional knockout mouse lines for the opioid and the cannabinoid systems.

*Target Gene*	Targeted neurons or structures for selective deletion “loss of function”	Targeted neurons or structures for selective expression “rescue”	Reference
***Opioid system***
*Oprm*	Primary sensory neurons expressing Nav1.8 channel (Nav1.8-Cre)		Weibel et al. (2013)
		Subpopulation of striatal medium spiny neurons	Cui et al. (2014)
*Oprd*	Primary sensory neurons expressing Nav1.8 channel (Nav1.8-Cre)		Gaveriaux-Ruff et al. (2011)
	Forebrain GABAergic neurons (Dlx5/6-Cre)		Chu Sin Chung et al. (2014)
*Oprk*	Dopamine containing neurons (DAT-Cre)		Van’t Veer et al. (2013)
***Cannabinoid system***
*Cnr1*	Principal forebrain neurons (CamKII-Cre)		Marsicano et al. (2003)
	Forebrain GABAergic neurons (Dlx5/6-Cre)		Monory et al. (2006)
	Cortical glutamatergic neurons (NEX-Cre)		Monory et al. (2006)
	Glutamatergic and GABAergic neurons (Glu/GABA)		Bellocchio et al. (2010)
	Primary sensory neurons expressing Nav1.8 channel (Nav1.8-Cre)		Agarwal et al. (2007)
	D1-dopaminergic neurons (Drd1a-Cre)		Monory et al. (2007)
	Serotoninergic neurons (TPH2-CreER^T2^)		Dubreucq et al. (2012b)
	Paraventricular hypothalamic neurons (Sim1-Cre)		Dubreucq et al. (2012a)
	Ventromedial hypothalamic neurons (SF1-cre)		Bellocchio et al. (2013)
	Neurons Nestin (Nes-Cre)		Maresz et al. (2007)
	Peripheral nerve (peripherin-Cre)		Pryce et al. (2014)
	Astrocytes (GFAP- CreER^T2^)		Han et al. (2012)
	Hepatocytes (Alb-Cre)		Jeong et al. (2008)
	Lymphocytes (lck-Cre)		Maresz et al. (2007)
	Keratinocytes (K14-Cre)		Gaffal et al. (2013)
		Dorsal telencephalic glutamatergic neurons (Glu-CB1-RS)	Ruehle et al. (2013)
*FAAH*		Nervous system (FAAH-NS)	Cravatt et al. (2004)

This table summarizes the recent published reports of cKO mouse lines for the different partners of opioid and cannabinoid systems using “loss of function” or “rescue” strategies.

In opposition to the loss of function approach, recent studies used a rescue strategy where the target gene is re-expressed in a null mutant, in only a subset of cells (**Table 2**). This helps to provide information concerning the sufficient role of the cell type expressing the target gene for a given function or establishing whether other cellular subtypes or circuits are necessary. When mu opioid receptor were re-expressed only in a subpopulation of striatal direct-pathway neurons, in a mu KO background, it restored opiate reward and opiate-induced striatal DA release, partially restored motivation to self-administer an opiate, but the rescued mice lacked opiate analgesia or withdrawal (Cui et al., 2014). In a similar genetic strategy, CB1 receptor expression was restored exclusively in dorsal telencephalic glutamatergic neurons and proved sufficient to control neuronal functions that are in large part hippocampus-dependent, while it was insufficient for proper amygdala functions (Ruehle et al., 2013). A conditional line where the expression of the FAAH enzyme has been restricted to the nervous system (FAAH-NS) was generated by crossing the FAAH KO line with a transgenic mouse, expressing FAAH under the neural specific enolase promoter (Cravatt et al., 2004). These mice exhibited a discrete subset of the biochemical and behavioral phenotypes observed in FAAH KO mice providing key insights into the distinct functions played by the central and peripheral lipids transmitter signaling systems **in vivo**.

In conclusion, despite potential limits such as developmental effects of the mutation or compensatory mechanisms to overcome consequences of the mutation, the use of mutants wherein a component of either opioid or cannabinoid system is selectively deleted from restricted neuronal populations provides essential tools for a comprehensive understanding of mechanisms underlying cannabinoid or opioid effects in reward circuitry. So far, these conditional lines for opioid and cannabinoid systems were mostly characterized for pain or emotional behavioral responses, and few data is yet to become available for reward aspects (**Table 2**).

## CANNABINOID REINFORCING EFFECTS IN OPIOID KNOCKOUT MICE

For evaluating the effect of cannabinoids in opioid mutant mice, THC-induced CPP was mostly used (**Table 3**). Interestingly, the same protocol was used for all tested opioid KO mice with 1 mg/kg ip dose with a priming injection in the home cage. In these conditions, no differences in place preference induced by THC was observed in delta or kappa KO mice while THC-CPP was abolished in mu KO mutants (Ghozland et al., 2002) as well as in the double mu-delta KO mice (Castane et al., 2003). These data support the hypothesis that mu receptors mediate rewarding properties of THC. A similar protocol was used to induce aversion, but with a higher dose of THC (5 mg/kg ip) wherein mu KO mice showed a decreased CPA (Ghozland et al., 2002). THC-induced CPA was abolished in similar conditions in both Pdyn (Zimmer et al., 2001) and kappa KO mice (Ghozland et al., 2002). Self-administration of the synthetic cannabinoid agonist WIN55,212-2 was successfully established in freely moving mice with a low priming dose (0.1 mg/kg i.p.,) and with this protocol, Pdyn KO mice showed facilitated SA (Mendizabal et al., 2006). Altogether, these data support the idea that the kappa/dynorphin system plays a key role in mediating cannabinoid dysphoric effects and therefore negatively modulates their rewarding effects (Mendizabal et al., 2006). Contribution of delta receptors in reward appears complex (Charbogne et al., 2014; Gutierrez-Cuesta et al., 2014) and it has not yet been established for cannabinoid reward, neither pharmacologically nor genetically. A potential role of this particular receptor in cannabinoid reward awaits further studies investigating either cannabinoid SA (motivation aspects) or delta cKO mutant responses (deletion of specific subpopulation of receptors).

**Table 3:** Rewarding and dependence responses for cannabinoids and opioids measured in KO mouse lines for both systems.

*Gene knockout*	Behavioral response	Genotype effect	Reference
***Opioid system***
*Oprm*	CPP, THC (1 mg/kg, i.p.)	Abolished	Ghozland et al. (2002)
	CPA THC (5 mg/kg, i.p.)	Decreased	
	WD, THC (20 mg/kg, i.p. 2x/d, 6d)	Unchanged	
	WD, THC (10 mg/kg, s.c. 5d)	Unchanged	Lichtman et al. (2001)
	WD, THC (30 or 100 mg/kg, s.c. 5d)	Decreased	
*Oprd*	CPP, THC (1 mg/kg, i.p.)	Unchanged	Ghozland et al. (2002)
	CPA THC (5 mg/kg, i.p.)	Unchanged	
	WD, THC (20 mg/kg, i.p. 2x/d, 6d)	Unchanged	
*Oprm/Oprd*	CPP, THC (1 mg/kg, i.p.)	Decreased	Castane et al. (2003)
	WD, THC (20 mg/kg, i.p. 2x/d, 6d)	Decreased	
*Oprk*	CPP, THC (1 mg/kg, i.p.)	Unchanged	Ghozland et al. (2002)
	CPP, THC (1 mg/kg, i.p.)w/o priming	Present, absent in WT	
	CPA, THC (5 mg/kg, i.p.)	Abolished	
	WD, THC (20 mg/kg, i.p. 2x/d, 6d)	Unchanged	
*Penk*	WD, THC (20 mg/kg, i.p. 2x/d, 6d)	Decreased	Valverde et al. (2000)
*Pdyn*	CPA, THC (5 mg/kg, i.p.)	Abolished	Ghozland et al. (2002)
	SA, WIN 55,212(6.25 mg/kg/inf, i.v.)	Increased	Mendizabal et al. (2006)
	SA, WIN 55,212(12.5 mg/kg/inf, i.v.)	Abolished	
	WD, THC (20 mg/kg, i.p. 2x/d, 6d)	Decreased	Zimmer et al. (2001)
***Cannabinoid system***
*Cnr1*	CPP, morphine (5 mg/kg, s.c.)	Abolished	Martin et al. (2000)
	CPA, morphine + naloxone (20–100 mg/kg i.p. over 6d + 0.1 mg/kg s.c.)	Unchanged	
	CPP, morphine (4–8 mg/kg, i.p.)	Unchanged	Rice et al. (2002)
	CPA, morphine + naloxone (8 mg/kg + 5 mg/kg, i.p.)	Unchanged	
	SA, morphine (2 ug/kg.inf, i.v.)	Abolished	Cossu et al. (2001)
	SA, morphine (1, 2, 4 ug/kg/inf, i.v.)	Decreased	Ledent et al. (1999)
	WD, morphine (20 mg/kg to 100 mg/kg, 5d)	Decreased	
	WD, morphine (75 mg/kg pellet, 5d)	Decreased	Lichtman et al. (2001)
	CPA, U50,488H (1 mg/kg, s.c.)	Abolished	Ledent et al. (1999)

This table summarizes published reports of behavioral responses in reward and precipitated withdrawal for cannabinoids in opioid KO lines and opioids in cannabinoid KO mutants (CPA, conditioned place aversion; CPP, conditioned place preference; d, day; inf, infusion; i.p., intraperitoneal; s.c., subcutaneous; WD, withdrawal; w/o, without).

Another aspect that was explored in opioid KO mice is cannabinoid dependence. Upon chronic THC treatment, antagonist-induced withdrawal signs measured in WT animals were unchanged for Pdyn KO (Zimmer et al., 2001) or single mutant mice for mu, delta or kappa opioid receptors (Ghozland et al., 2002). Signs were attenuated in KO animals for Penk (Valverde et al., 2000), for the double mu-delta receptor mutant (Castane et al., 2003) as well as for mu receptor KO, at a high dose (Lichtman et al., 2001) (**Table 3**). No data are yet available for the other opioid peptide KO mice concerning cannabinoid physical dependence. Collectively, available data indicate the involvement of the enkephalinergic system, with a cooperative action of mu and delta receptors, in the expression of cannabinoid dependence.

## OPIOID REINFORCING EFFECTS IN CANNABINOID KNOCKOUT MICE

Knockout approaches have greatly improved our knowledge on the role of CB1 receptors in addiction in general, even though contradictory data exist (Maldonado et al., 2006). In particular, for opiate responses (**Table 3**) induced by mu agonists, CB1 KO mice showed no morphine-induced place preference (5 mg/kg, s.c., 3 injections over 6 days) (Martin et al., 2000) and a diminished propensity to self-administer morphine (Ledent et al., 1999; Cossu et al., 2001). A microdialysis study revealed that morphine-induced increase of extracellular DA was not observed in CB1 KO mice (Mascia et al., 1999). Taken together, these data suggest a reduction in morphine’s reinforcing activity in the absence of the CB1 receptor. Another study could not reveal any changes in place preference using a slightly more intensive conditioning paradigm and a different set up with two doses of morphine (4 or 8 mg/kg, four injections over 4 days) (Rice et al., 2002). Interestingly, no differences between WT and CB1 KO mice could be observed in a CPA paradigm where the opioid antagonist naloxone was used to induce withdrawal in morphine-treated mice via two distinct paradigms (Martin et al., 2000; Rice et al., 2002). Upon chronic morphine treatment, naloxone-induced withdrawal signs measured in WT animals were attenuated (Ledent et al., 1999; Lichtman et al., 2001). Together, these findings suggest that CB1 receptors are not involved in the disphoric effects of morphine withdrawal (CPA) but are noticeably required for the development of physical dependence or of somatic signs of opiate withdrawal. Surprisingly, other important effects of morphine, like acute induced analgesia and tolerance to chronic morphine-induced analgesia, were not altered in CB1 KO animals. These findings together with the data on mu opioid KO mice with cannabinoid treatments suggest a bidirectional influence of mu opioid and CB1 cannabinoid receptors on reward processes. Aversive effects of the kappa opioid agonist U50,488H were also blunted in CB1 receptor KO mice (Ledent et al., 1999). Together with the data of kappa opioid and Pdyn KO mice, it indicates that both cannabinoid and opioid systems modulate negative motivational drug effects. To our knowledge, no data concerning the specific effect of delta selective opioid agonists on reward in CB1 KO mice are available. Interestingly, it has been demonstrated that the absence of CB1 receptor also results in a reduction of the sensitivity to the rewarding properties of sucrose (Sanchis-Segura et al., 2004), as well as other reinforcers (for recent reviews, see (Lopez-Moreno et al., 2010; Maldonado et al., 2013). Together with pharmacological approaches (Maldonado et al., 2006), KO data therefore provide confirmatory support that CB1 receptor play a modulatory role in the reinforced behaviors maintained by sucrose and some other reinforcers with, in particular, a mutual interaction of opioid and cannabinoid systems.

For the other components of the endocannabinoid system, no specific data for genetically modified animals were reported for the investigation of opioid reward, although pharmacological inhibition of the endocannabinoid catabolic enzymes attenuates both naloxone-induced withdrawal as well as spontaneous withdrawal signs in morphine dependent mice (Ramesh et al., 2011, 2013), indicating a potential role of these enzymes in opioid dependence.

## CONCLUSION AND PERSPECTIVES

Globally, despite some compensatory alterations at both opioid and cannabinoid levels in mutant lines, KO studies have provided insights into the mutual role of both opioid and cannabinoid systems on reward. In particular, these studies have highlighted the major role for both mu opioid and CB1 receptors in these processes. Clearly, the mu opioid receptor is a convergent molecular target mediating rewarding properties of opioid compounds but also of other drugs of abuse, including cannabinoids. CB1 receptor also appears as a modulator of opioid reward. On the other hand, KO approaches for endogenous opioid peptides or enzymes for synthesis or degradation of endocannabinoids have been very useful to clarify their specific role in both endogenous systems but less/no data are available for reward mechanisms. These mutants therefore need further investigations to clarify their potential implication in cannabinoid/opioid reward aspects.

Conventional genetically modified animals have strengthened our current knowledge of the interaction between these two systems, but further studies using conditional approaches will be necessary to clarify the potential crosstalk existing specifically in reward processes. Interaction between these two neuromodulator systems may be dependent on the brain area where it occurs, even inside the brain rewarding networks (Parolaro et al., 2010). Both mu opioid and CB1 receptors are highly expressed in these networks in similar brain structures and a potential interaction in areas where they are both strongly expressed is probable. Noticeably, opposite expression levels are observed in discrete areas like amygdala (BLA versus central amygdala) as well as habenula (medial versus lateral nuclei) and these differences may also account for a modulatory role of the two systems in reward processes (**Figure 1**). Approaches using double mutants for both receptors would be useful to further understand their mutual role in drug reward. Moreover, in this perspective, conditional approaches will surely provide invaluable insights into opioid and cannabinoid interaction at the circuitry level. The growing number of cKO mutant lines becoming available will help this side of research. Likewise, the implication of the CB2 receptor in these interactions has not yet been explored and may be particularly relevant in specific brain structures. In fact, demonstration of CB2 expression in several brain structures has opened a field of investigation for a possible role in addiction that should help to reveal potential direct interaction between CB2 and the opioid system.

G protein coupled receptor can associate as heteromers and extended research is now directed toward elucidating the physiological role of such heteromers and finding therapeutical approaches targeting these entities (see recent reviews Fujita et al., 2014; Massotte, 2014). Several lines of evidence have suggested interactions between delta or mu opioid receptors and the CB1 receptors. Close vicinity of CB1 receptors with mu or delta opioid receptors has recently been established at the neuronal level, suggesting heteromeric formation *in vivo* and potential impact on both receptors signaling properties. A recent study demonstrated an important role for the heterodimer CB1-delta in neuropathic pain where cortical functions of delta receptor were altered (Bushlin et al., 2012). CB1 and mu receptors associate as heteromers in cultured cells and a recent study showed that bivalent ligands for both receptors are potent analgesic devoid of tolerance (Le Naour et al., 2013), suggesting potential functional heteromers in pain. Therefore, one can easily predict that similar mechanisms may occur in another pathological state like addiction and this opens up new prospects for pharmacological action of cannabinoid and opioid drugs. In this context, it will be critical to see whether CB2 also plays a role as a potential heteromeric interactor with opioid receptors.

No effective therapeutic approaches for cannabis dependence are currently available and opioid addiction therapies are not fully satisfying for all patients. Further studies are therefore needed to clarify the mechanistic basis of interaction of the two systems, which would aid in the development of drug therapies to reduce dependence and abuse. Antibodies or bivalent ligands as mentioned previously represent interesting therapeutic targets. In addition, dual enkephalinase inhibitors and cannabinoid catabolic enzyme inhibitors have been proposed as attractive therapeutic targets to treat pain (Roques et al., 2012) and such bi-functional compounds may also be relevant as promising strategies for alleviating dependence.

Substantial progress has been made in understanding the cellular and molecular mechanisms of prolonged use of cannabinoid or opioid drugs (Kreek et al., 2012; Fratta and Fattore, 2013). In addition to their direct role in reward, interaction between opioid and cannabinoid neuromodulator systems has been proposed to explain some aspects of vulnerability to addiction and, in this perspective, recent attention has been focused on yet another critical level, epigenetics. These molecular processes, including methylation of DNA, post-translational modifications of histones and regulation by microRNA, regulate gene expression and are crucial in long-term adaptations induced by drugs (Nestler, 2014). Recent studies have shown a direct association between THC-induced Penk upregulation through reduction of histone H3 lysine 9 pattern of methylation and increased heroin SA (Tomasiewicz et al., 2012). Adolescent THC-exposure also resulted in altered heroin SA in the subsequent generation of rats, an effect associated with changes in mRNA expression of cannabinoid, DA, and glutamatergic receptor genes in the striatum, suggesting adaptations to long-term drug effect and germline transmission, most likely involving epigenetic changes (Szutorisz et al., 2014). How these neuromodulator systems are dependent on various internal and external environmental factors, and therefore are involved in epigenetics and whether one system influences the epigenetic machinery to control the other system, are unresolved questions for upcoming studies (D’Addario et al., 2013). Future investigation in this field will be necessary to better delineate the neurobiological mechanisms underlying these neuroadaptations.

## Conflict of Interest Statement

The author declares that the research was conducted in the absence of any commercial or financial relationships that could be construed as a potential conflict of interest.

## ABBREVIATIONS

2-AG: 2-arachidonoylglycerol
AEA: anandamide, N-arachidonoylethanolamide
CB1: type 1 cannabinoid receptor
CB2: type 2 cannabinoid receptor
cKO: conditional knockout mice
CPA: conditioned place aversion
CP 55,940: (1R,3R,4R)-3-[2-hydroxy-4-(1,1-dimethylheptyl)phenyl]-4-(3-hydroxypropyl)cyclohexan-1-ol
CPP: conditioned place preference
CPu: caudate putamen
DA: dopamine
DAGL: diacylglycerol lipase
FAAH: fatty acid amide hydrolase
G protein: guanine nucleotide binding protein
GABA: c-aminobutyric acid
GPCR: G protein coupled receptor
KO: knockout
MGL: monoacylglycerol lipase
NAPE-PLD: N-acyl phosphatidylethanolamine phospholipase D
THC: Delta-9-tetrahydrocannabinol
WIN 55,212-2: 2,3-dihydro-5-methyl-3-[(morpholinyl)methyl]pyrrolo[1,2,3-de]-1,4-benzoxazin-yl-1-naphtalenylmethanone mesylate
WT: wild-type

## References

[B1] Aaltonen N. Riera Ribas C. Lehtonen M. Savinainen J. R. Laitinen J. T. (2014). Brain regional cannabinoid CB(1) receptor signalling and alternative enzymatic pathways for 2-arachidonoylglycerol generation in brain sections of diacylglycerol lipase deficient mice. *Eur. J. Pharm. Sci.* 51 87–95 10.1016/j.ejps.2013.08.035 24012970

[B2] Agarwal N. Pacher P. Tegeder I. Amaya F. Constantin C. E. Brenner G. J. (2007). Cannabinoids mediate analgesia largely via peripheral type 1 cannabinoid receptors in nociceptors. *Nat. Neurosci.* 10 870–879 10.1038/nn1916 17558404 PMC2234438

[B3] Ahn K. Mckinney M. K. Cravatt B. F. (2008). Enzymatic pathways that regulate endocannabinoid signaling in the nervous system. *Chem. Rev.* 108 1687–1707 10.1021/cr0782067 18429637 PMC3150828

[B4] Ansonoff M. A. Zhang J. Czyzyk T. Rothman R. B. Stewart J. Xu H. (2006). Antinociceptive and hypothermic effects of salvinorin a are abolished in a novel strain of kappa-opioid receptor-1 knockout mice. *J. Pharmacol. Exp. Ther.* 318 641–648 10.1124/jpet.106.101998 16672569

[B5] Belin D. Deroche-Gamonet V. (2012). Responses to novelty and vulnerability to cocaine addiction: contribution of a multi-symptomatic animal model. *Cold Spring Harb. Perspect. Med.* 2:a011940 10.1101/cshperspect.a011940. PMC3543096 23125204

[B6] Bellocchio L. Lafenetre P. Cannich A. Cota D. Puente N. Grandes P. (2010). Bimodal control of stimulated food intake by the endocannabinoid system. *Nat. Neurosci.* 13 281–283 10.1038/nn.2494 20139974

[B7] Bellocchio L. Soria-Gomez E. Quarta C. Metna-Laurent M. Cardinal P. Binder E. (2013). Activation of the sympathetic nervous system mediates hypophagic and anxiety-like effects of CB(1) receptor blockade. *Proc. Natl. Acad. Sci. U.S.A.* 110 4786–4791 10.1073/pnas.1218573110 23487769 PMC3607008

[B8] Berrendero F. Mendizabal V. Murtra P. Kieffer B. L. Maldonado R. (2003). Cannabinoid receptor and WIN 55 212-2-stimulated [35S]-GTPgammaS binding in the brain of mu-, delta-, and kappa-opioid receptor knockout mice. *Eur. J. Neurosci.* 18 2197–2202 10.1046/j.1460-9568.2003.02951.x 14622180

[B9] Bodnar R. J. (2014). Endogenous opiates and behavior: 2013. *Peptides 62C* 67–136 10.1016/j.peptides.2014.09.013 25263178

[B10] Brady L. S. Herkenham M. Rothman R. B. Partilla J. S. Konig M. Zimmer A. M. (1999). Region-specific up-regulation of opioid receptor binding in enkephalin knockout mice. *Brain Res. Mol. Brain Res.* 68 193–197 10.1016/S0169-328X(99)00090-X 10320798

[B11] Buckley N. E. Mccoy K. L. Mezey E. Bonner T. Zimmer A. Felder C. C. (2000). Immunomodulation by cannabinoids is absent in mice deficient for the cannabinoid CB(2) receptor. *Eur. J. Pharmacol.* 396 141–149 10.1016/S0014-2999(00)00211-9 10822068

[B12] Bushlin I. Gupta A. Stockton S. D. Jr. Miller L. K. Devi L. A. (2012). Dimerization with cannabinoid receptors allosterically modulates delta opioid receptor activity during neuropathic pain. *PLoS ONE* 7:e49789 10.1371/journal.pone.0049789 PMC3522681 23272051

[B13] Castane A. Robledo P. Matifas A. Kieffer B. L. Maldonado R. (2003). Cannabinoid withdrawal syndrome is reduced in double mu and delta opioid receptor knockout mice. *Eur. J. Neurosci.* 17 155–159 10.1046/j.1460-9568.2003.02409.x 12534979

[B14] Chanda P. K. Gao Y. Mark L. Btesh J. Strassle B. W. Lu P. (2010). Monoacylglycerol lipase activity is a critical modulator of the tone and integrity of the endocannabinoid system. *Mol. Pharmacol.* 78 996–1003 10.1124/mol.110.068304 20855465

[B15] Charbogne P. Kieffer B. L. Befort K. (2014). 15 years of genetic approaches in vivo for addiction research: opioid receptor and peptide gene knockout in mouse models of drug abuse. *Neuropharmacology* 76(Pt B) 204–217 10.1016/j.neuropharm.2013.08.028 24035914 PMC3858501

[B16] Chu Sin Chung P. Keyworth H. L. Martin-Garcia E. Charbogne P. Darcq E. Bailey A. (2014). A novel anxiogenic role for the delta opioid receptor expressed in GABAergic forebrain neurons. *Biol. Psychiatry* 10.1016/j.biopsych.2014.07.033 [Epub ahead of print]. PMC4297504 25444168

[B17] Clarke S. Czyzyk T. Ansonoff M. Nitsche J. F. Hsu M. S. Nilsson L. (2002). Autoradiography of opioid and ORL1 ligands in opioid receptor triple knockout mice. *Eur. J. Neurosci.* 16 1705–1712 10.1046/j.1460-9568.2002.02239.x 12431223

[B18] Clarke S. Zimmer A. Zimmer A. M. Hill R. G. Kitchen I. (2003). Region selective up-regulation of micro-, delta- and kappa-opioid receptors but not opioid receptor-like 1 receptors in the brains of enkephalin and dynorphin knockout mice. *Neuroscience* 122 479–489 10.1016/j.neuroscience.2003.07.011 14614912

[B19] Contet C. Kieffer B. L. Befort K. (2004). Mu opioid receptor: a gateway to drug addiction. *Curr. Opin. Neurobiol.* 14 370–378 10.1016/j.conb.2004.05.005 15194118

[B20] Cossu G. Ledent C. Fattore L. Imperato A. Bohme G. A. Parmentier M. (2001). Cannabinoid CB1 receptor knockout mice fail to self-administer morphine but not other drugs of abuse. *Behav. Brain Res.* 118 61–65 10.1016/S0166-4328(00)00311-9 11163634

[B21] Cravatt B. F. Demarest K. Patricelli M. P. Bracey M. H. Giang D. K. Martin B. R. (2001). Supersensitivity to anandamide and enhanced endogenous cannabinoid signaling in mice lacking fatty acid amide hydrolase. *Proc. Natl. Acad. Sci. U.S.A.* 98 9371–9376 10.1073/pnas.161191698 11470906 PMC55427

[B22] Cravatt B. F. Giang D. K. Mayfield S. P. Boger D. L. Lerner R. A. Gilula N. B. (1996). Molecular characterization of an enzyme that degrades neuromodulatory fatty-acid amides. *Nature* 384 83–87 10.1038/384083a0 8900284

[B23] Cravatt B. F. Saghatelian A. Hawkins E. G. Clement A. B. Bracey M. H. Lichtman A. H. (2004). Functional disassociation of the central and peripheral fatty acid amide signaling systems. *Proc. Natl. Acad. Sci. U.S.A.* 101 10821–10826 10.1073/pnas.0401292101 15247426 PMC490018

[B24] Cui Y. Ostlund S. B. James A. S. Park C. S. Ge W. Roberts K. W. (2014). Targeted expression of mu-opioid receptors in a subset of striatal direct-pathway neurons restores opiate reward. *Nat. Neurosci.* 17 254–261 10.1038/nn.3622 24413699 PMC4008330

[B25] D’Addario C. Di Francesco A. Pucci M. Finazzi Agro A. Maccarrone M. (2013). Epigenetic mechanisms and endocannabinoid signalling. *FEBS J.* 280 1905–1917 10.1111/febs.12125 23305292

[B26] De Luca M. A. Valentini V. Bimpisidis Z. Cacciapaglia F. Caboni P. Di Chiara G. (2014). Endocannabinoid 2-arachidonoylglycerol self-administration by sprague-dawley rats and stimulation of in vivo dopamine transmission in the nucleus accumbens Shell. *Front. Psychiatry* 5:140 10.3389/fpsyt.2014.00140 PMC4201088 25368584

[B27] De Petrocellis L. Di Marzo V. (2010). Non-CB1, non-CB2 receptors for endocannabinoids, plant cannabinoids, and synthetic cannabimimetics: focus on G-protein-coupled receptors and transient receptor potential channels. *J. Neuroimmune Pharmacol.* 5 103–121 10.1007/s11481-009-9177-z 19847654

[B28] Dhopeshwarkar A. Mackie K. (2014). CB2 cannabinoid receptors as a therapeutic target-what does the future hold? *Mol. Pharmacol*. 86 430–437 10.1124/mol.114.094649 25106425 PMC4164977

[B29] Di Marzo V. Breivogel C. S. Tao Q. Bridgen D. T. Razdan R. K. Zimmer A. M. (2000). Levels, metabolism, and pharmacological activity of anandamide in CB(1) cannabinoid receptor knockout mice: evidence for non-CB(1), non-CB(2) receptor-mediated actions of anandamide in mouse brain. *J. Neurochem.* 75 2434–2444 10.1046/j.1471-4159.2000.0752434.x 11080195

[B30] Dinh T. P. Carpenter D. Leslie F. M. Freund T. F. Katona I. Sensi S. L. (2002). Brain monoglyceride lipase participating in endocannabinoid inactivation. *Proc. Natl. Acad. Sci. U.S.A.* 99 10819–10824 10.1073/pnas.152334899 12136125 PMC125056

[B31] Dubreucq S. Kambire S. Conforzi M. Metna-Laurent M. Cannich A. Soria-Gomez E. (2012a). Cannabinoid type 1 receptors located on single-minded 1-expressing neurons control emotional behaviors. *Neuroscience* 204 230–244 10.1016/j.neuroscience.2011.08.049 21920410

[B32] Dubreucq S. Matias I. Cardinal P. Haring M. Lutz B. Marsicano G. (2012b). Genetic dissection of the role of cannabinoid type-1 receptors in the emotional consequences of repeated social stress in mice. *Neuropsychopharmacology* 37 1885–1900 10.1038/npp.2012.36 22434220 PMC3376321

[B33] Erbs E. Faget L. Scherrer G. Matifas A. Filliol D. Vonesch J. L. (2014). A mu-delta opioid receptor brain atlas reveals neuronal co-occurrence in subcortical networks. *Brain Struct. Funct.* 10.1007/s00429-014-0717-9 [Epub ahead of print]. PMC4341027 24623156

[B34] Evans C. J. Keith D. E. Jr. Morrison H. Magendzo K. Edwards R. H. (1992). Cloning of a delta opioid receptor by functional expression. *Science* 258 1952–1955 10.1126/science.1335167 1335167

[B35] Everitt B. J. Robbins T. W. (2005). Neural systems of reinforcement for drug addiction: from actions to habits to compulsion. *Nat. Neurosci.* 8 1481–1489 10.1038/nn1579 16251991

[B36] Fattore L. Cossu G. Martellotta C. M. Fratta W. (2001). Intravenous self-administration of the cannabinoid CB1 receptor agonist WIN 55,212-2 in rats. *Psychopharmacology (Berl)* 156 410–416 10.1007/s002130100734 11498718

[B37] Fattore L. Deiana S. Spano S. M. Cossu G. Fadda P. Scherma M. (2005). Endocannabinoid system and opioid addiction: behavioural aspects. *Pharmacol. Biochem. Behav.* 81 343–359 10.1016/j.pbb.2005.01.031 15935459

[B38] Fattore L. Fratta W. (2011). Beyond THC: the new generation of cannabinoid designer drugs. *Front. Behav. Neurosci.* 5:60 10.3389/fnbeh.2011.00060 PMC3187647 22007163

[B39] Fernandez-Ruiz J. Hernandez M. Ramos J. A. (2010). Cannabinoid-dopamine interaction in the pathophysiology and treatment of CNS disorders. *CNS Neurosci. Ther.* 16 e72–e91 10.1111/j.1755-5949.2010.00144.x 20406253 PMC6493786

[B40] Ferre S. Lluis C. Justinova Z. Quiroz C. Orru M. Navarro G. (2010). Adenosine-cannabinoid receptor interactions. Implications for striatal function. *Br. J. Pharmacol.* 160 443–453 10.1111/j.1476-5381.2010.00723.x 20590556 PMC2931547

[B41] Filizola M. Devi L. A. (2014). Grand opening of structure-guided design for novel opioids. *Trends Pharmacol. Sci.* 34 6–12 10.1016/j.tips.2012.10.002 23127545 PMC3534797

[B42] Filliol D. Ghozland S. Chluba J. Martin M. Matthes H. W. Simonin F. (2000). Mice deficient for delta- and mu-opioid receptors exhibit opposing alterations of emotional responses. *Nat. Genet.* 25 195–200 10.1038/76061 10835636

[B43] Flores A. Maldonado R. Berrendero F. (2014). The hypocretin/orexin receptor-1 as a novel target to modulate cannabinoid reward. *Biol. Psychiatry* 75 499–507 10.1016/j.biopsych.2013.06.012 23896204

[B44] Fowler C. D. Kenny P. J. (2012). Utility of genetically modified mice for understanding the neurobiology of substance use disorders. *Hum. Genet.* 131 941–957 10.1007/s00439-011-1129-z 22190154 PMC3977433

[B45] Fratta W. Fattore L. (2013). Molecular mechanisms of cannabinoid addiction. *Curr. Opin. Neurobiol.* 23 487–492 10.1016/j.conb.2013.02.002 23490548

[B46] Frazier C. J. (2011). Key questions of endocannabinoid signalling in the CNS: which, where and when? *J. Physiol*. 589 4807–4808 10.1113/jphysiol.2011.219493 22001725 PMC3224874

[B47] Fujita W. Gomes I. Devi L. A. (2014). Revolution in GPCR signalling: opioid receptor heteromers as novel therapeutic targets: IUPHAR review 10. *Br. J. Pharmacol.* 171 4155–4176 10.1111/bph.12798 24916280 PMC4241085

[B48] Gaffal E. Cron M. Glodde N. Bald T. Kuner R. Zimmer A. (2013). Cannabinoid 1 receptors in keratinocytes modulate proinflammatory chemokine secretion and attenuate contact allergic inflammation. *J. Immunol.* 190 4929–4936 10.4049/jimmunol.1201777 23585676

[B49] Gao Y. Vasilyev D. V. Goncalves M. B. Howell F. V. Hobbs C. Reisenberg M. (2010). Loss of retrograde endocannabinoid signaling and reduced adult neurogenesis in diacylglycerol lipase knock-out mice. *J. Neurosci.* 30 2017–2024 10.1523/JNEUROSCI.5693-09.2010 20147530 PMC6634037

[B50] Gardner E. L. (2005). Endocannabinoid signaling system and brain reward: emphasis on dopamine. *Pharmacol. Biochem. Behav.* 81 263–284 10.1016/j.pbb.2005.01.032 15936806

[B51] Gaveriaux-Ruff C. (2013). Opiate-induced analgesia: contributions from mu, delta and kappa opioid receptors mouse mutants. *Curr. Pharm. Des* 19 7373–7381 10.2174/138161281942140105163727 23448470

[B52] Gaveriaux-Ruff C. Nozaki C. Nadal X. Hever X. C. Weibel R. Matifas A. (2011). Genetic ablation of delta opioid receptors in nociceptive sensory neurons increases chronic pain and abolishes opioid analgesia. *Pain* 152 1238–1248 10.1016/j.pain.2010.12.031 21295407

[B53] Gerald T. M. Howlett A. C. Ward G. R. Ho C. Franklin S. O. (2008). Gene expression of opioid and dopamine systems in mouse striatum: effects of CB1 receptors, age and sex. *Psychopharmacology (Berl.)* 198 497–508 10.1007/s00213-008-1141-1148 18438728 PMC3708653

[B54] Gerald T. M. Ward G. R. Howlett A. C. Franklin S. O. (2006). CB1 knockout mice display significant changes in striatal opioid peptide and D4 dopamine receptor gene expression. *Brain Res.* 1093 20–24 10.1016/j.brainres.2006.03.088 16684513

[B55] Ghozland S. Matthes H. W. Simonin F. Filliol D. Kieffer B. L. Maldonado R. (2002). Motivational effects of cannabinoids are mediated by mu-opioid and kappa-opioid receptors. *J. Neurosci.* 22 1146–1154. 11826143 10.1523/JNEUROSCI.22-03-01146.2002 PMC6758535

[B56] Gong J. P. Onaivi E. S. Ishiguro H. Liu Q. R. Tagliaferro P. A. Brusco A. (2006). Cannabinoid CB2 receptors: immunohistochemical localization in rat brain. *Brain Res.* 1071 10–23 10.1016/j.brainres.2005.11.035 16472786

[B57] Goody R. J. Oakley S. M. Filliol D. Kieffer B. L. Kitchen I. (2002). Quantitative autoradiographic mapping of opioid receptors in the brain of delta-opioid receptor gene knockout mice. *Brain Res.* 945 9–19 10.1016/S0006-8993(02)02452-6 12113946

[B58] Gustavsson A. Svensson M. Jacobi F. Allgulander C. Alonso J. Beghi E. (2011). Cost of disorders of the brain in Europe 2010. *Eur. Neuropsychopharmacol.* 21 718–779 10.1016/j.euroneuro.2011.08.008 21924589

[B59] Gutierrez-Cuesta J. Burokas A. Mancino S. Kummer S. Martin-Garcia E. Maldonado R. (2014). Effects of genetic deletion of endogenous opioid system components on the reinstatement of cocaine-seeking behavior in mice. *Neuropsychopharmacology.* 10.1038/npp.2014.149 PMC4229567 24943644

[B60] Han J. Kesner P. Metna-Laurent M. Duan T. Xu L. Georges F. (2012). Acute cannabinoids impair working memory through astroglial CB1 receptor modulation of hippocampal LTD. *Cell* 148 1039–1050 10.1016/j.cell.2012.01.037 22385967

[B61] Hough L. B. Nalwalk J. W. Chen Y. Schuller A. Zhu Y. Zhang J. (2000). Improgan, a cimetidine analog, induces morphine-like antinociception in opioid receptor-knockout mice. *Brain Res.* 880 102–108 10.1016/S0006-8993(00)02776-1 11032994

[B62] Ignatowska-Jankowska B. M. Muldoon P. P. Lichtman A. H. Damaj M. I. (2013). The cannabinoid CB2 receptor is necessary for nicotine-conditioned place preference, but not other behavioral effects of nicotine in mice. *Psychopharmacology (Berl)* 229 591–601 10.1007/s00213-013-3117-6 23652588 PMC4042856

[B63] Jarai Z. Wagner J. A. Varga K. Lake K. D. Compton D. R. Martin B. R. (1999). Cannabinoid-induced mesenteric vasodilation through an endothelial site distinct from CB1 or CB2 receptors. *Proc. Natl. Acad. Sci. U.S.A.* 96 14136–14141 10.1073/pnas.96.24.14136 10570211 PMC24203

[B64] Jeong W. I. Osei-Hyiaman D. Park O. Liu J. Batkai S. Mukhopadhyay P. (2008). Paracrine activation of hepatic CB1 receptors by stellate cell-derived endocannabinoids mediates alcoholic fatty liver. *Cell Metab.* 7 227–235 10.1016/j.cmet.2007.12.007 18316028

[B65] Kieffer B. L. (1995). Recent advances in molecular recognition and signal transduction of active peptides: receptors for opioid peptides. *Cell Mol. Neurobiol.* 15 615–635 10.1007/BF02071128 8719033 PMC11563145

[B66] Kieffer B. L. Befort K. Gaveriaux-Ruff C. Hirth C. G. (1992). The delta-opioid receptor: isolation of a cDNA by expression cloning and pharmacological characterization. *Proc. Natl. Acad. Sci. U.S.A.* 89 12048–12052 10.1073/pnas.89.24.12048 1334555 PMC50695

[B67] Kieffer B. L. Gaveriaux-Ruff C. (2002). Exploring the opioid system by gene knockout. *Prog. Neurobiol.* 66 285–306 10.1016/S0301-0082(02)00008-4 12015197

[B68] Kitchen I. Slowe S. J. Matthes H. W. Kieffer B. (1997). Quantitative autoradiographic mapping of mu-, delta-, and kappa-opioid receptors in knockout mice lacking the mu-opioid receptor gene. *Brain Res.* 778 73–88 10.1016/S0006-8993(97)00988-8 9462879

[B69] Konig M. Zimmer A. M. Steiner H. Holmes P. V. Crawley J. N. Brownstein M. J. (1996). Pain responses, anxiety and aggression in mice deficient in pre-proenkephalin. *Nature* 383 535–538 10.1038/383535a0 8849726

[B70] Koob G. F. (2009). Neurobiological substrates for the dark side of compulsivity in addiction. *Neuropharmacology* 56(Suppl. 1) 18–31 10.1016/j.neuropharm.2008.07.043 18725236 PMC2637927

[B71] Koob G. F. Volkow N. D. (2010). Neurocircuitry of addiction. *Neuropsychopharmacology* 35 217–238 10.1038/npp.2009.110 19710631 PMC2805560

[B72] Kreek M. J. Levran O. Reed B. Schlussman S. D. Zhou Y. Butelman E. R. (2012). Opiate addiction and cocaine addiction: underlying molecular neurobiology and genetics. *J. Clin. Invest.* 122 3387–3393 10.1172/JCI60390 23023708 PMC3534165

[B73] Le Merrer J. Becker J. A. Befort K. Kieffer B. L. (2009). Reward processing by the opioid system in the brain. *Physiol. Rev.* 89 1379–1412 10.1152/physrev.00005.2009 19789384 PMC4482114

[B74] Le Naour M. Akgun E. Yekkirala A. Lunzer M. M. Powers M. D. Kalyuzhny A. E. (2013). Bivalent ligands that target mu opioid (MOP) and cannabinoid1 (CB1) receptors are potent analgesics devoid of tolerance. *J. Med. Chem.* 56 5505–5513 10.1021/jm4005219 23734559 PMC3849126

[B75] Ledent C. Valverde O. Cossu G. Petitet F. Aubert J. F. Beslot F. (1999). Unresponsiveness to cannabinoids and reduced addictive effects of opiates in CB1 receptor knockout mice. *Science* 283 401–404 10.1126/science.283.5400.401 9888857

[B76] Leshner A. I. (1997). Addiction is a brain disease, and it matters. *Science* 278 45–47 10.1126/science.278.5335.45 9311924

[B77] Leung D. Saghatelian A. Simon G. M. Cravatt B. F. (2006). Inactivation of N-acyl phosphatidylethanolamine phospholipase D reveals multiple mechanisms for the biosynthesis of endocannabinoids. *Biochemistry* 45 4720–4726 10.1021/bi060163l 16605240 PMC1538545

[B78] Lichtman A. H. Sheikh S. M. Loh H. H. Martin B. R. (2001). Opioid and cannabinoid modulation of precipitated withdrawal in delta(9)-tetrahydrocannabinol and morphine-dependent mice. *J. Pharmacol. Exp. Ther.* 298 1007–1014. 11504797

[B79] Liu J. Wang L. Harvey-White J. Huang B. X. Kim H. Y. Luquet S. (2008). Multiple pathways involved in the biosynthesis of anandamide. *Neuropharmacology* 54 1–7 10.1016/j.neuropharm.2007.05.020 17631919 PMC2219543

[B80] Loacker S. Sayyah M. Wittmann W. Herzog H. Schwarzer C. (2007). Endogenous dynorphin in epileptogenesis and epilepsy: anticonvulsant net effect via kappa opioid receptors. *Brain* 130 1017–1028 10.1093/brain/awl384 17347252

[B81] Loh H. H. Liu H. C. Cavalli A. Yang W. Chen Y. F. Wei L. N. (1998). mu Opioid receptor knockout in mice: effects on ligand-induced analgesia and morphine lethality. *Brain Res. Mol. Brain Res.* 54 321–326 10.1016/S0169-328X(97)00353-7 9555078

[B82] Lopez-Moreno J. A. Lopez-Jimenez A. Gorriti M. A. De Fonseca F. R. (2010). Functional interactions between endogenous cannabinoid and opioid systems: focus on alcohol, genetics and drug-addicted behaviors. *Curr. Drug Targets* 11 406–428 10.2174/138945010790980312 20196742

[B83] Lutz P. E. Ayranci G. Chu-Sin-Chung P. Matifas A. Koebel P. Filliol D. (2014). Distinct mu, delta, and kappa opioid receptor mechanisms underlie low sociability and depressive-like behaviors during heroin abstinence. *Neuropsychopharmacology* 39 2694–2705 10.1038/npp.2014.126 24874714 PMC4207349

[B84] Lutz P. E. Kieffer B. L. (2013a). The multiple facets of opioid receptor function: implications for addiction. *Curr. Opin. Neurobiol.* 23 473–479 10.1016/j.conb.2013.02.005 23453713 PMC3702666

[B85] Lutz P. E. Kieffer B. L. (2013b). Opioid receptors: distinct roles in mood disorders. *Trends Neurosci.* 36 195–206 10.1016/j.tins.2012.11.002 23219016 PMC3594542

[B86] Maccarrone M. Attina M. Bari M. Cartoni A. Ledent C. Finazzi-Agro A. (2001). Anandamide degradation and N-acylethanolamines level in wild-type and CB1 cannabinoid receptor knockout mice of different ages. *J. Neurochem.* 78 339–348 10.1046/j.1471-4159.2001.00413.x 11461969

[B87] Maccarrone M. Valverde O. Barbaccia M. L. Castane A. Maldonado R. Ledent C. (2002). Age-related changes of anandamide metabolism in CB1 cannabinoid receptor knockout mice: correlation with behaviour. *Eur. J. Neurosci.* 15 1178–1186 10.1046/j.1460-9568.2002.01957.x 11982628

[B88] Mackie K. (2005). Distribution of cannabinoid receptors in the central and peripheral nervous system. *Handb. Exp. Pharmacol.* 299–325. 16596779 10.1007/3-540-26573-2_10

[B89] Mackie K. (2008). Cannabinoid receptors: where they are and what they do. *J. Neuroendocrinol.* 20(Suppl. 1) 10–14 10.1111/j.1365-2826.2008.01671.x 18426493

[B90] Maldonado R. (2002). Study of cannabinoid dependence in animals. *Pharmacol. Ther.* 95 153–164 10.1016/S0163-7258(02)00254-1 12182962

[B91] Maldonado R. Berrendero F. Ozaita A. Robledo P. (2011). Neurochemical basis of cannabis addiction. *Neuroscience* 181 1–17 10.1016/j.neuroscience.2011.02.035 21334423

[B92] Maldonado R. Blendy J. A. Tzavara E. Gass P. Roques B. P. Hanoune J. (1996). Reduction of morphine abstinence in mice with a mutation in the gene encoding CREB. *Science* 273 657–659 10.1126/science.273.5275.657 8662559

[B93] Maldonado R. Robledo P. Berrendero F. (2013). Endocannabinoid system and drug addiction: new insights from mutant mice approaches. *Curr. Opin. Neurobiol.* 23 480–486 10.1016/j.conb.2013.02.004 23490550

[B94] Maldonado R. Valverde O. Berrendero F. (2006). Involvement of the endocannabinoid system in drug addiction. *Trends Neurosci.* 29 225–232 10.1016/j.tins.2006.01.008 16483675

[B95] Mansour A. Fox C. A. Akil H. Watson S. J. (1995). Opioid-receptor mRNA expression in the rat CNS: anatomical and functional implications. *Trends Neurosci.* 18 22–29 10.1016/0166-2236(95)93946-U 7535487

[B96] Manzanares J. Corchero J. Romero J. Fernandez-Ruiz J. J. Ramos J. A. Fuentes J. A. (1999). Pharmacological and biochemical interactions between opioids and cannabinoids. *Trends Pharmacol. Sci.* 20 287–294 10.1016/S0165-6147(99)01339-5 10390647

[B97] Maresz K. Pryce G. Ponomarev E. D. Marsicano G. Croxford J. L. Shriver L. P. (2007). Direct suppression of CNS autoimmune inflammation via the cannabinoid receptor CB1 on neurons and CB2 on autoreactive T cells. *Nat. Med.* 13 492–497 10.1038/nm1561 17401376

[B98] Marsicano G. Goodenough S. Monory K. Hermann H. Eder M. Cannich A. (2003). CB1 cannabinoid receptors and on-demand defense against excitotoxicity. *Science* 302 84–88 10.1126/science.1088208 14526074

[B99] Marsicano G. Wotjak C. T. Azad S. C. Bisogno T. Rammes G. Cascio M. G. (2002). The endogenous cannabinoid system controls extinction of aversive memories. *Nature* 418 530–534 10.1038/nature00839 12152079

[B100] Martellotta M. C. Cossu G. Fattore L. Gessa G. L. Fratta W. (1998). Self-administration of the cannabinoid receptor agonist WIN 55,212-2 in drug-naive mice. *Neuroscience* 85 327–330 10.1016/S0306-4522(98)00052-9 9622233

[B101] Martin M. Ledent C. Parmentier M. Maldonado R. Valverde O. (2000). Cocaine, but not morphine, induces conditioned place preference and sensitization to locomotor responses in CB1 knockout mice. *Eur. J. Neurosci.* 12 4038–4046 10.1046/j.1460-9568.2000.00287.x 11069600

[B102] Mascia M. S. Obinu M. C. Ledent C. Parmentier M. Bohme G. A. Imperato A. (1999). Lack of morphine-induced dopamine release in the nucleus accumbens of cannabinoid CB(1) receptor knockout mice. *Eur. J. Pharmacol* 383 R1–R2 10.1016/S0014-2999(99)00656-1 10594337

[B103] Massotte D. (2014). In vivo opioid receptor heteromerization: where do we stand? *Br. J. Pharmacol*. 172 420–434 10.1111/bph.12702 24666391 PMC4292957

[B104] Matsuda L. A. Lolait S. J. Brownstein M. J. Young A. C. Bonner T. I. (1990). Structure of a cannabinoid receptor and functional expression of the cloned cDNA. *Nature* 346 561–564 10.1038/346561a0 2165569

[B105] Matthes H. W. Maldonado R. Simonin F. Valverde O. Slowe S. Kitchen I. (1996). Loss of morphine-induced analgesia, reward effect and withdrawal symptoms in mice lacking the mu-opioid-receptor gene. *Nature* 383 819–823 10.1038/383819a0 8893006

[B106] Mendizabal V. Zimmer A. Maldonado R. (2006). Involvement of kappa/dynorphin system in WIN 55,212-2 self-administration in mice. *Neuropsychopharmacology* 31 1957–1966 10.1038/sj.npp.1300957 16292318

[B107] Mestek A. Hurley J. H. Bye L. S. Campbell A. D. Chen Y. Tian M. (1995). The human mu opioid receptor: modulation of functional desensitization by calcium/calmodulin-dependent protein kinase and protein kinase C. *J. Neurosci.* 15 2396–2406. 7891175 10.1523/JNEUROSCI.15-03-02396.1995 PMC6578163

[B108] Mizoguchi H. Watanabe C. Osada S. Yoshioka M. Aoki Y. Natsui S. (2010). Lack of a rewarding effect and a locomotor-enhancing effect of the selective mu-opioid receptor agonist amidino-TAPA. *Psychopharmacology (Berl)* 212 215–225 10.1007/s00213-010-1946-0 20683583

[B109] Monory K. Blaudzun H. Massa F. Kaiser N. Lemberger T. Schutz G. (2007). Genetic dissection of behavioural and autonomic effects of Delta(9)-tetrahydrocannabinol in mice. *PLoS Biol.* 5:e269 10.1371/journal.pbio.0050269 PMC2001214 17927447

[B110] Monory K. Massa F. Egertova M. Eder M. Blaudzun H. Westenbroek R. (2006). The endocannabinoid system controls key epileptogenic circuits in the hippocampus. *Neuron* 51 455–466 10.1016/j.neuron.2006.07.006 16908411 PMC1769341

[B111] Munro S. Thomas K. L. Abu-Shaar M. (1993). Molecular characterization of a peripheral receptor for cannabinoids. *Nature* 365 61–65 10.1038/365061a0 7689702

[B112] Nadal X. La Porta C. Andreea Bura S. Maldonado R. (2013). Involvement of the opioid and cannabinoid systems in pain control: new insights from knockout studies. *Eur. J. Pharmacol.* 716 142–157 10.1016/j.ejphar.2013.01.077 23523475

[B113] Navarrete F. Rodriguez-Arias M. Martin-Garcia E. Navarro D. Garcia-Gutierrez M. S. Aguilar M. A. (2013). Role of CB2 cannabinoid receptors in the rewarding, reinforcing, and physical effects of nicotine. *Neuropsychopharmacology* 38 2515–2524 10.1038/npp.2013.157 23817165 PMC3799072

[B114] Nestler E. J. (2014). Epigenetic mechanisms of drug addiction. *Neuropharmacology* 76 Pt B, 259–268 10.1016/j.neuropharm.2013.04.004 23643695 PMC3766384

[B115] Niikura K. Narita M. Okutsu D. Tsurukawa Y. Nanjo K. Kurahashi K. (2008). Implication of endogenous beta-endorphin in the inhibition of the morphine-induced rewarding effect by the direct activation of spinal protein kinase C in mice. *Neurosci. Lett.* 433 54–58 10.1016/j.neulet.2007.12.042 18262361

[B116] Nogueiras R. Romero-Pico A. Vazquez M. J. Novelle M. G. Lopez M. Dieguez C. (2014). The opioid system and food intake: homeostatic and hedonic mechanisms. *Obes. Facts* 5 196–207 10.1159/000338163 22647302

[B117] Oakley S. M. Toth G. Borsodi A. Kieffer B. L. Kitchen I. (2003). G-protein coupling of delta-opioid receptors in brains of mu-opioid receptor knockout mice. *Eur. J. Pharmacol.* 466 91–98 10.1016/S0014-2999(03)01531-0 12679145

[B118] Ohno-Shosaku T. Kano M. (2014). Endocannabinoid-mediated retrograde modulation of synaptic transmission. *Curr. Opin. Neurobiol.* 29C 1–8 10.1016/j.conb.2014.03.017 24747340

[B119] Onaivi E. S. Ishiguro H. Gong J. P. Patel S. Perchuk A. Meozzi P. A. (2006). Discovery of the presence and functional expression of cannabinoid CB2 receptors in brain. *Ann. N. Y. Acad. Sci.* 1074 514–536 10.1196/annals.1369.052 17105950

[B120] Ortega-Alvaro A. Ternianov A. Aracil-Fernandez A. Navarrete F. Garcia-Gutierrez M. S. Manzanares J. (2013). Role of cannabinoid CB receptor in the reinforcing actions of ethanol. *Addict. Biol.* 10.1111/adb.12076 23855434

[B121] Pan Y. X. Xu J. Xu M. Rossi G. C. Matulonis J. E. Pasternak G. W. (2009). Involvement of exon 11-associated variants of the mu opioid receptor MOR-1 in heroin, but not morphine, actions. *Proc. Natl. Acad. Sci. U.S.A.* 106 4917–4922 10.1073/pnas.0811586106 19273844 PMC2660730

[B122] Panagis G. Mackey B. Vlachou S. (2014). Cannabinoid regulation of brain reward processing with an emphasis on the role of CB1 receptors: a step back into the future. *Front. Psychiatry* 5:92 10.3389/fpsyt.2014.00092 PMC4117180 25132823

[B123] Panlilio L. V. Goldberg S. R. (2007). Self-administration of drugs in animals and humans as a model and an investigative tool. *Addiction* 102 1863–1870 10.1111/j.1360-0443.2007.02011.x 18031422 PMC2695138

[B124] Panlilio L. V. Justinova Z. Goldberg S. R. (2010). Animal models of cannabinoid reward. *Br. J. Pharmacol.* 160 499–510 10.1111/j.1476-5381.2010.00775.x 20590560 PMC2931551

[B125] Parolaro D. Rubino T. Vigano D. Massi P. Guidali C. Realini N. (2010). Cellular mechanisms underlying the interaction between cannabinoid and opioid system. *Curr. Drug Targets* 11 393–405 10.2174/138945010790980367 20017730

[B126] Pattij T. De Vries T. J. (2013). The role of impulsivity in relapse vulnerability. *Curr. Opin. Neurobiol.* 23 700–705 10.1016/j.conb.2013.01.023 23462336

[B127] Pertwee R. G. (2010). Receptors and channels targeted by synthetic cannabinoid receptor agonists and antagonists. *Curr. Med. Chem* 17 1360–1381 10.2174/092986710790980050 20166927 PMC3013229

[B128] Petrenko A. B. Yamazaki M. Sakimura K. Kano M. Baba H. (2014). Augmented tonic pain-related behavior in knockout mice lacking monoacylglycerol lipase, a major degrading enzyme for the endocannabinoid 2-arachidonoylglycerol. *Behav. Brain Res.* 271 51–58 10.1016/j.bbr.2014.05.063 24906199

[B129] Piomelli D. (2014). More surprises lying ahead. The endocannabinoids keep us guessing. *Neuropharmacology* 76 Pt B 228–234 10.1016/j.neuropharm.2013.07.026 23954677 PMC3855347

[B130] Pol O. Murtra P. Caracuel L. Valverde O. Puig M. M. Maldonado R. (2006). Expression of opioid receptors and c-fos in CB1 knockout mice exposed to neuropathic pain. *Neuropharmacology* 50 123–132 10.1016/j.neuropharm.2005.11.002 16360182

[B131] Pradhan A. A. Befort K. Nozaki C. Gaveriaux-Ruff C. Kieffer B. L. (2011). The delta opioid receptor: an evolving target for the treatment of brain disorders. *Trends Pharmacol. Sci.* 32 581–590 10.1016/j.tips.2011.06.008 21925742 PMC3197801

[B132] Pryce G. Visintin C. Ramagopalan S. V. Al-Izki S. De Faveri L. E. Nuamah R. A. (2014). Control of spasticity in a multiple sclerosis model using central nervous system-excluded CB1 cannabinoid receptor agonists. *FASEB J.* 28 117–130 10.1096/fj.13-239442 24121462

[B133] Ragnauth A. Schuller A. Morgan M. Chan J. Ogawa S. Pintar J. (2001). Female preproenkephalin-knockout mice display altered emotional responses. *Proc. Natl. Acad. Sci. U.S.A.* 98 1958–1963 10.1073/pnas.041598498 11172058 PMC29364

[B134] Ramesh D. Gamage T. F. Vanuytsel T. Owens R. A. Abdullah R. A. Niphakis M. J. (2013). Dual inhibition of endocannabinoid catabolic enzymes produces enhanced antiwithdrawal effects in morphine-dependent mice. *Neuropsychopharmacology* 38 1039–1049 10.1038/npp.2012.269 23303065 PMC3629394

[B135] Ramesh D. Ross G. R. Schlosburg J. E. Owens R. A. Abdullah R. A. Kinsey S. G. (2011). Blockade of endocannabinoid hydrolytic enzymes attenuates precipitated opioid withdrawal symptoms in mice. *J. Pharmacol. Exp. Ther.* 339 173–185 10.1124/jpet.111.181370 21719468 PMC3186294

[B136] Rice O. V. Gordon N. Gifford A. N. (2002). Conditioned place preference to morphine in cannabinoid CB1 receptor knockout mice. *Brain Res.* 945 135–138 10.1016/S0006-8993(02)02890-1 12113961

[B137] Robbe D. Kopf M. Remaury A. Bockaert J. Manzoni O. J. (2002). Endogenous cannabinoids mediate long-term synaptic depression in the nucleus accumbens. *Proc. Natl. Acad. Sci. U.S.A.* 99 8384–8388 10.1073/pnas.122149199 12060781 PMC123076

[B138] Robinson T. E. Berridge K. C. (2008). Review. The incentive sensitization theory of addiction: some current issues. *Philos. Trans. R. Soc. Lond. B Biol. Sci.* 363 3137–3146 10.1098/rstb.2008.0093 18640920 PMC2607325

[B139] Robledo P. Berrendero F. Ozaita A. Maldonado R. (2008). Advances in the field of cannabinoid–opioid cross-talk. *Addict. Biol.* 13 213–224 10.1111/j.1369-1600.2008.00107.x 18482431

[B140] Roques B. P. Fournie-Zaluski M. C. Wurm M. (2012). Inhibiting the breakdown of endogenous opioids and cannabinoids to alleviate pain. *Nat. Rev. Drug Discov.* 11 292–310 10.1038/nrd3673 22460123

[B141] Rubinstein M. Mogil J. S. Japon M. Chan E. C. Allen R. G. Low M. J. (1996). Absence of opioid stress-induced analgesia in mice lacking beta-endorphin by site-directed mutagenesis. *Proc. Natl. Acad. Sci. U.S.A.* 93 3995–4000 10.1073/pnas.93.9.3995 8633004 PMC39474

[B142] Ruehle S. Remmers F. Romo-Parra H. Massa F. Wickert M. Wortge S. (2013). Cannabinoid CB1 receptor in dorsal telencephalic glutamatergic neurons: distinctive sufficiency for hippocampus-dependent and amygdala-dependent synaptic and behavioral functions. *J. Neurosci.* 33 10264–10277 10.1523/JNEUROSCI.4171-12.2013 23785142 PMC6618598

[B143] Sanchis-Segura C. Cline B. H. Marsicano G. Lutz B. Spanagel R. (2004). Reduced sensitivity to reward in CB1 knockout mice. *Psychopharmacology (Berl)* 176 223–232 10.1007/s00213-004-1877-1878 15083252

[B144] Sanchis-Segura C. Spanagel R. (2006). Behavioural assessment of drug reinforcement and addictive features in rodents: an overview. *Addict. Biol.* 11 2–38 10.1111/j.1369-1600.2006.00012.x 16759333

[B145] Saunders B. T. Robinson T. E. (2013). Individual variation in resisting temptation: implications for addiction. *Neurosci. Biobehav. Rev.* 37 1955–1975 10.1016/j.neubiorev.2013.02.008 23438893 PMC3732519

[B146] Scavone J. L. Sterling R. C. Van Bockstaele E. J. (2013). Cannabinoid and opioid interactions: implications for opiate dependence and withdrawal. *Neuroscience* 248 637–654 10.1016/j.neuroscience.2013.04.034 23624062 PMC3742578

[B147] Schlosburg J. E. Blankman J. L. Long J. Z. Nomura D. K. Pan B. Kinsey S. G. (2010). Chronic monoacylglycerol lipase blockade causes functional antagonism of the endocannabinoid system. *Nat. Neurosci.* 13 1113–1119 10.1038/nn.2616 20729846 PMC2928870

[B148] Schuller A. G. King M. A. Zhang J. Bolan E. Pan Y. X. Morgan D. J. (1999). Retention of heroin and morphine-6 beta-glucuronide analgesia in a new line of mice lacking exon 1 of MOR-1. *Nat. Neurosci.* 2 151–156 10.1038/5706 10195199

[B149] Sharifi N. Diehl N. Yaswen L. Brennan M. B. Hochgeschwender U. (2001). Generation of dynorphin knockout mice. *Brain Res. Mol. Brain Res.* 86 70–75 10.1016/S0169-328X(00)00264-3 11165373

[B150] Simonin F. Befort K. Gaveriaux-Ruff C. Matthes H. Nappey V. Lannes B. (1994). The human delta-opioid receptor: genomic organization, cDNA cloning, functional expression, and distribution in human brain. *Mol. Pharmacol.* 46 1015–1021. 7808419

[B151] Simonin F. Gaveriaux-Ruff C. Befort K. Matthes H. Lannes B. Micheletti G. (1995). kappa-Opioid receptor in humans: cDNA and genomic cloning, chromosomal assignment, functional expression, pharmacology, and expression pattern in the central nervous system. *Proc. Natl. Acad. Sci. U.S.A.* 92 7006–7010 10.1073/pnas.92.15.7006 7624359 PMC41460

[B152] Simonin F. Slowe S. Becker J. A. Matthes H. W. Filliol D. Chluba J. (2001). Analysis of [3H]bremazocine binding in single and combinatorial opioid receptor knockout mice. *Eur. J. Pharmacol.* 414 189–195 10.1016/S0014-2999(01)00822-6 11239918

[B153] Simonin F. Valverde O. Smadja C. Slowe S. Kitchen I. Dierich A. (1998). Disruption of the kappa-opioid receptor gene in mice enhances sensitivity to chemical visceral pain, impairs pharmacological actions of the selective kappa-agonist U-50,488H and attenuates morphine withdrawal. *EMBO J.* 17 886–897 10.1093/emboj/17.4.886 9463367 PMC1170438

[B154] Skoubis P. D. Lam H. A. Shoblock J. Narayanan S. Maidment N. T. (2005). Endogenous enkephalins, not endorphins, modulate basal hedonic state in mice. *Eur. J. Neurosci.* 21 1379–1384 10.1111/j.1460-9568.2005.03956.x 15813947

[B155] Slowe S. J. Simonin F. Kieffer B. Kitchen I. (1999). Quantitative autoradiography of mu-delta- and kappa1 opioid receptors in kappa-opioid receptor knockout mice. *Brain Res.* 818 335–345 10.1016/S0006-8993(98)01201-3 10082819

[B156] Solinas M. Goldberg S. R. Piomelli D. (2008). The endocannabinoid system in brain reward processes. *Br. J. Pharmacol.* 154 369–383 10.1038/bjp.2008.130 18414385 PMC2442437

[B157] Sora I. Elmer G. Funada M. Pieper J. Li X. F. Hall F. S. (2001). Mu opiate receptor gene dose effects on different morphine actions: evidence for differential in vivo mu receptor reserve. *Neuropsychopharmacology* 25 41–54 10.1016/S0893-133X(00)00252-9 11377918

[B158] Stein C. (2013). Opioids, sensory systems and chronic pain. *Eur. J. Pharmacol.* 716 179–187 10.1016/j.ejphar.2013.01.076 23500206

[B159] Steiner H. Bonner T. I. Zimmer A. M. Kitai S. T. Zimmer A. (1999). Altered gene expression in striatal projection neurons in CB1 cannabinoid receptor knockout mice. *Proc. Natl. Acad. Sci. U.S.A.* 96 5786–5790 10.1073/pnas.96.10.5786 10318962 PMC21938

[B160] Sun X. Wang H. Okabe M. Mackie K. Kingsley P. J. Marnett L. J. (2009). Genetic loss of faah compromises male fertility in mice. *Biol. Reprod.* 80 235–242 10.1095/biolreprod.108.072736 18987328 PMC2804815

[B161] Swendsen J. Le Moal M. (2011). Individual vulnerability to addiction. *Ann. N. Y. Acad. Sci.* 1216 73–85 10.1111/j.1749-6632.2010.05894.x 21272012

[B162] Szutorisz H. Dinieri J. A. Sweet E. Egervari G. Michaelides M. Carter J. M. (2014). Parental THC exposure leads to compulsive heroin-seeking and altered striatal synaptic plasticity in the subsequent generation. *Neuropsychopharmacology* 39 1315–1323 10.1038/npp.2013.352 24385132 PMC3988557

[B163] Tanimura A. Yamazaki M. Hashimotodani Y. Uchigashima M. Kawata S. Abe M. (2010). The endocannabinoid 2-arachidonoylglycerol produced by diacylglycerol lipase alpha mediates retrograde suppression of synaptic transmission. *Neuron* 65 320–327 10.1016/j.neuron.2010.01.021 20159446

[B164] Taschler U. Radner F. P. Heier C. Schreiber R. Schweiger M. Schoiswohl G. (2011). Monoglyceride lipase deficiency in mice impairs lipolysis and attenuates diet-induced insulin resistance. *J. Biol. Chem.* 286 17467–17477 10.1074/jbc.M110.215434 21454566 PMC3093820

[B165] Tebano M. T. Martire A. Popoli P. (2012). Adenosine A(2A)-cannabinoid CB(1) receptor interaction: an integrative mechanism in striatal glutamatergic neurotransmission. *Brain Res.* 1476 108–118 10.1016/j.brainres.2012.04.051 22565012

[B166] Tian M. Broxmeyer H. E. Fan Y. Lai Z. Zhang S. Aronica S. (1997). Altered hematopoiesis, behavior, and sexual function in mu opioid receptor-deficient mice. *J. Exp. Med.* 185 1517–1522 10.1084/jem.185.8.1517 9126934 PMC2196276

[B167] Tomasiewicz H. C. Jacobs M. M. Wilkinson M. B. Wilson S. P. Nestler E. J. Hurd Y. L. (2012). Proenkephalin mediates the enduring effects of adolescent cannabis exposure associated with adult opiate vulnerability. *Biol. Psychiatry* 72 803–810 10.1016/j.biopsych.2012.04.026 22683090 PMC3440551

[B168] Trigo J. M. Martin-Garcia E. Berrendero F. Robledo P. Maldonado R. (2010). The endogenous opioid system: a common substrate in drug addiction. *Drug Alcohol Depend.* 108 183–194 10.1016/j.drugalcdep.2009.10.011 19945803

[B169] Tsuboi K. Okamoto Y. Ikematsu N. Inoue M. Shimizu Y. Uyama T. (2011). Enzymatic formation of N-acylethanolamines from N-acylethanolamine plasmalogen through N-acylphosphatidylethanolamine-hydrolyzing phospholipase D-dependent and -independent pathways. *Biochim. Biophys. Acta* 1811 565–577 10.1016/j.bbalip.2011.07.009 21801852

[B170] Tzschentke T. M. (2007). Measuring reward with the conditioned place preference (CPP) paradigm: update of the last decade. *Addict. Biol.* 12 227–462 10.1111/j.1369-1600.2007.00070.x 17678505

[B171] Uchigashima M. Yamazaki M. Yamasaki M. Tanimura A. Sakimura K. Kano M. (2011). Molecular and morphological configuration for 2-arachidonoylglycerol-mediated retrograde signaling at mossy cell-granule cell synapses in the dentate gyrus. *J. Neurosci.* 31 7700–7714 10.1523/JNEUROSCI.5665-10.2011 21613483 PMC6633146

[B172] Uriguen L. Berrendero F. Ledent C. Maldonado R. Manzanares J. (2005). Kappa- and delta-opioid receptor functional activities are increased in the caudate putamen of cannabinoid CB1 receptor knockout mice. *Eur. J. Neurosci.* 22 2106–2110 10.1111/j.1460-9568.2005.04372.x 16262648

[B173] Valverde O. Maldonado R. Valjent E. Zimmer A. M. Zimmer A. (2000). Cannabinoid withdrawal syndrome is reduced in pre-proenkephalin knock-out mice. *J. Neurosci.* 20 9284–9289. 11125007 10.1523/JNEUROSCI.20-24-09284.2000 PMC6773016

[B174] Valverde O. Torrens M. (2012). CB1 receptor-deficient mice as a model for depression. *Neuroscience* 204 193–206 10.1016/j.neuroscience.2011.09.031 21964469

[B175] Van’t Veer A. Bechtholt A. J. Onvani S. Potter D. Wang Y. Liu-Chen L. Y. (2013). Ablation of kappa-opioid receptors from brain dopamine neurons has anxiolytic-like effects and enhances cocaine-induced plasticity. *Neuropsychopharmacology* 38 1585–1597 10.1038/npp.2013.58 23446450 PMC3682153

[B176] van Rijn R. M. Whistler J. L. (2009). The delta(1) opioid receptor is a heterodimer that opposes the actions of the delta(2) receptor on alcohol intake. *Biol. Psychiatry* 66 777–784 10.1016/j.biopsych.2009.05.019 19576572 PMC2757485

[B177] Vigano D. Rubino T. Parolaro D. (2005). Molecular and cellular basis of cannabinoid and opioid interactions. *Pharmacol. Biochem. Behav.* 81 360–368 10.1016/j.pbb.2005.01.021 15927245

[B178] Vinod K. Y. Sanguino E. Yalamanchili R. Manzanares J. Hungund B. L. (2008). Manipulation of fatty acid amide hydrolase functional activity alters sensitivity and dependence to ethanol. *J. Neurochem.* 104 233–243 10.1111/j.1471-4159.2007.04956.x 17944864

[B179] Weibel R. Reiss D. Karchewski L. Gardon O. Matifas A. Filliol D. (2013). Mu opioid receptors on primary afferent nav1.8 neurons contribute to opiate-induced analgesia: insight from conditional knockout mice. *PLoS ONE* 8:e74706 10.1371/journal.pone.0074706 PMC3771900 24069332

[B180] Wise L. E. Shelton C. C. Cravatt B. F. Martin B. R. Lichtman A. H. (2007). Assessment of anandamide’s pharmacological effects in mice deficient of both fatty acid amide hydrolase and cannabinoid CB1 receptors. *Eur. J. Pharmacol.* 557 44–48 10.1016/j.ejphar.2006.11.002 17217945

[B181] Wotherspoon G. Fox A. Mcintyre P. Colley S. Bevan S. Winter J. (2005). Peripheral nerve injury induces cannabinoid receptor 2 protein expression in rat sensory neurons. *Neuroscience* 135 235–245 10.1016/j.neuroscience.2005.06.009 16084654

[B182] Xi Z. X. Peng X. Q. Li X. Song R. Zhang H. Y. Liu Q. R. (2011). Brain cannabinoid CB(2) receptors modulate cocaine’s actions in mice. *Nat. Neurosci.* 14 1160–1166 10.1038/nn.2874 21785434 PMC3164946

[B183] Yaswen L. Diehl N. Brennan M. B. Hochgeschwender U. (1999). Obesity in the mouse model of pro-opiomelanocortin deficiency responds to peripheral melanocortin. *Nat. Med.* 5 1066–1070 10.1038/12506 10470087

[B184] Yoshino H. Miyamae T. Hansen G. Zambrowicz B. Flynn M. Pedicord D. (2011). Postsynaptic diacylglycerol lipase mediates retrograde endocannabinoid suppression of inhibition in mouse prefrontal cortex. *J. Physiol.* 589 4857–4884 10.1113/jphysiol.2011.212225 21807615 PMC3224880

[B185] Zanettini C. Panlilio L. V. Alicki M. Goldberg S. R. Haller J. Yasar S. (2011). Effects of endocannabinoid system modulation on cognitive and emotional behavior. *Front. Behav. Neurosci.* 5:57 10.3389/fnbeh.2011.00057 PMC3171696 21949506

[B186] Zhang H. Y. Gao M. Liu Q. R. Bi G. H. Li X. Yang H. J. (2014). Cannabinoid CB2 receptors modulate midbrain dopamine neuronal activity and dopamine-related behavior in mice. *Proc. Natl. Acad. Sci. U.S.A.* 10.1073/pnas.1413210111 PMC4246322 25368177

[B187] Zhu Y. King M. A. Schuller A. G. Nitsche J. F. Reidl M. Elde R. P. (1999). Retention of supraspinal delta-like analgesia and loss of morphine tolerance in delta opioid receptor knockout mice. *Neuron* 24 243–252 10.1016/S0896-6273(00)80836-3 10677041

[B188] Zimmer A. Valjent E. Konig M. Zimmer A. M. Robledo P. Hahn H. (2001). Absence of delta-9-tetrahydrocannabinol dysphoric effects in dynorphin-deficient mice. *J. Neurosci.* 21 9499–9505. 11717384 10.1523/JNEUROSCI.21-23-09499.2001 PMC6763924

[B189] Zimmer A. Zimmer A. M. Hohmann A. G. Herkenham M. Bonner T. I. (1999). Increased mortality, hypoactivity, and hypoalgesia in cannabinoid CB1 receptor knockout mice. *Proc. Natl. Acad. Sci. U.S.A.* 96 5780–5785 10.1073/pnas.96.10.5780 10318961 PMC21937

